# The Effect of Arbuscular Mycorrhizal Fungus and Phosphorus Treatment on Root Metabolome of *Medicago lupulina* During Key Stages of Development

**DOI:** 10.3390/plants14172685

**Published:** 2025-08-28

**Authors:** Andrey P. Yurkov, Roman K. Puzanskiy, Alexey A. Kryukov, Tatyana R. Kudriashova, Anastasia I. Kovalchuk, Anastasia I. Gorenkova, Ekaterina M. Bogdanova, Yuri V. Laktionov, Daria A. Romanyuk, Vladislav V. Yemelyanov, Alexey L. Shavarda, Maria F. Shishova

**Affiliations:** 1 Laboratory of Ecology of Symbiotic and Associative Rhizobacteria, All-Russia Research Institute for Agricultural Microbiology, Pushkin, St. Petersburg 196608, Russia; aa.krukov@arriam.ru (A.A.K.); t.kudryashova@arriam.ru (T.R.K.); a.kovalchuk@arriam.ru (A.I.K.); nastya.gorenkova.2016@mail.ru (A.I.G.); laktionov@arriam.ru (Y.V.L.); d.romanyuk@arriam.ru (D.A.R.); 2 Laboratory of Analytical Phytochemistry, Komarov Botanical Institute of the Russian Academy of Sciences, St. Petersburg 197022, Russia; puzansky@yandex.ru (R.K.P.); bogdanova.ekaterina15@gmail.com (E.M.B.); stachyopsis@gmail.com (A.L.S.); 3 Graduate School of Biotechnology and Food Science, Peter the Great St. Petersburg Polytechnic University, St. Petersburg 194064, Russia; 4 Faculty of Biology, St. Petersburg State University, St. Petersburg 199034, Russia; bootika@mail.ru (V.V.Y.); mshishova@mail.ru (M.F.S.); 5 Center for Molecular and Cell Technologies, St. Petersburg State University, St. Petersburg 199034, Russia

**Keywords:** plant–fungal symbiosis, metabolic profile, GC-MS, plant development, Pi deficiency, biochemical networks, *Medicago lupulina*, *Rhizophagus irregularis*

## Abstract

The arbuscular mycorrhizal fungi (AMF) effect on the plant metabolome is an actual question of plant biology. Its alteration during host plant development and at different phosphorus supplies is of special interest. The aim of this study was to evaluate the effect of *Rhizophagus irregularis* (Błaszk., Wubet, Renker & Buscot) C. Walker & A. Schüßler inoculation and/or phosphorus treatment on the root metabolome of *Medicago lupulina* L. subsp. *vulgaris* Koch at the first true leaf, second leaf, third leaf development stages, the lateral branching initiation, the flowering and the mature fruit stages. The assessment of metabolic profiles was performed using GC-MS. In total, 327 metabolites were annotated: among them 20 carboxylic acids, 26 amino acids, 14 fatty acids and 58 sugars. The efficient AM was characterized by the upregulation of the metabolism of proteins, carbohydrates and lipids, as well as an increase in the content of phosphates. The tricarboxylic acid abundance was generally lower during mycorrhization. Fourteen metabolic markers of the efficient AM symbiosis were identified. The lateral branching initiation stage was shown to have key importance. Long-lasting metabolomic profiling indicated variances in mycorrhization and Pi supply effects at different key stages of host plant development.

## 1. Introduction

Phosphorus, along with nitrogen, is the main macronutrient for plants [1]. However 80% of soil phosphorus is immobile and unavailable for plant uptake due to conversion to organic form, precipitation or adsorption [2]. Meanwhile, plants actively absorb the forms of inorganic phosphorus (Pi) available for nutrition: HPO_4_^2−^, H_3_PO_4_ and, mainly, H_2_PO_4_^−^. Moreover, phosphorus mobilization is possible due to the development of proteoid roots in phosphorus deficiency conditions [3]. Such roots produce a significant amount of organic acids and other metabolites (up to 23% of photosynthates) with different functions, such as acidification of the soil and chelation of metal ions. This mechanism allows plants to absorb only 8% of the Pi from the rhizosphere [1]. The most powerful machinery to supply plant organisms with phosphorus is the symbiotic pathway represented by the development of arbuscular mycorrhiza (AM) [4].

Arbuscular mycorrhizal fungi (AMF) are gathered in the only class of Glomeromycetes of the monophyletic division Glomeromycota [5]. They form a symbiosis with more than 80% of terrestrial plant species [4]. Symbiosis with AMF allows plants to adapt to low levels of Pi in the soil via several ways: (1) AMF mycelium increases the absorbent surface of the root 100-fold [4]; (2) AMF helps the host plant to overcome the depletion zone with a low Pi content within a radius of 1–2 mm from the root surface [6]; (3) the diameter of mycorrhizosphere formed by the host plant and AMF reaches 10 to 150 mm around the root [7]; (4) in Pi deficiency conditions, macronutrient uptake into the plant is mainly carried out through phosphate transporters of the AMF, for example, RiPT7 in *Rhizophagus irregularis* (Błaszk., Wubet, Renker & Buscot) C. Walker & A. Schüßler [8], the elevation of plant phosphate transporters [9] and H^+^-ATPase [10] expression in AM roots; (5) AMF secretes organic acids [11] and their own phosphatases [12], which allow plants to reduce the resources required for phosphatase activity [13]. Thus, the contribution of inorganic phosphorus uptake by AMF through hyphae and arbuscules can reach 90–99% of the total demand of the host plant [14]. Such diverse processes cannot proceed without corresponding changes at the systemic level, which are actively studied using approaches such as transcriptomics [15], proteomics [16] and metabolomics [17]. However, at present, the mechanisms of plant adaptation to low Pi levels in the soil have not been fully discovered [8].

A number of studies focused on the analysis of metabolic rearrangements of the host plant during mycorrhization. The mechanisms of adaptation to low Pi level were performed on such model plants as *Medicago truncatula* Gaertn. [18,19], *Anadenanthera colubrina* Speg. [20] and *Solanum lycopersicum* L. [21]. The majority of studies contain only an analysis of the vegetation stages of plant development, without evaluation of the reproductive stages or vice versa. Long-term metabolic studies of plant–AMF symbiosis are very rare [22,23,24,25,26]. Identification of the most significant effect of AMF on the metabolome of the host plant was provided under conditions of low Pi levels in the following plant microbial systems (PMS): “*M. lupulina* L. + *R. irregularis*” [24]; “*Sorghum caudatum* (Hack.) Stapf/*S. bicolor* (L.) Moench + *R. irregularis*/*Gigaspora gigantea* (T.H.Nicolson & Gerd.) Gerd. & Trappe” [27]; “*Malus domestica* (Suckow) Borkh. + *R. irregularis*” [27]; “*Coffea arabica* L. + *Funnelliformis mosseae* (T.H. Nicolson & Gerd.) C. Walker & A. Schüßler” [28]. Some studies were carried out under conditions of high or optimal Pi levels in PMS such as “*Triticum durum* Desf. + mixture of strains of nine AMF species, including *R. irregularis*” with a weak response to mycorrhization [29]. A promising area of research is the analysis of PMS development in conditions of an optimal Pi level in the substrate. Such conditions can be considered as controls, since the lack of Pi is a stress factor that has its own effect on the metabolic profile. Thus, the number of studies related to the development of mycorrhizal plants under conditions of different levels of Pi is deficient and the mechanisms of adaptation of mycorrhizal plants to the lack of Pi in the substrate are not fully revealed [8,13,30].

It can be hypothesized that only complex long-lasting metabolic investigation will be able to reveal peculiarities of plant biochemical adaptation to phosphorus deficiency with and without symbiosis with AMF. These would be more distinguished with the highly effective *M. lupulina* line. The aim of this study was to evaluate the effect of *R. irregularis* inoculation and phosphorus treatment on the root metabolome of the *M. lupulina* MlS-1 line throughout key vegetative and reproductive stages from the first leaf development to the mature fruit.

## 2. Results

### 2.1. Plant Development Under Different Phosphorus Levels and AMF Treatment

**Figure 1:**
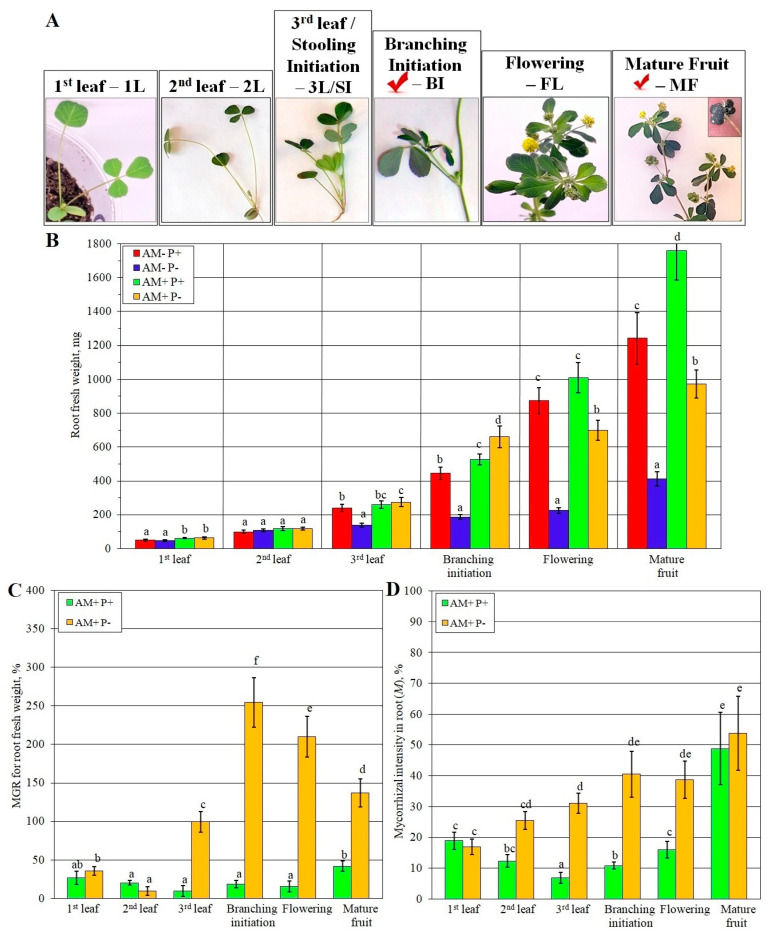
Key stages of *Medicago lupulina* plant development (**A**), fresh weight of roots (**B**), mycorrhizal growth response for root fresh weight (**C**) per one *M. lupulina* plant inoculated with *R. irregularis*, mycorrhizal intensity, *M* (**D**). Symbol: red tick indicates important developmental stages. “a”, “b”, “c” et al.—different letters indicate significant differences within the same MGR parameter (ANOVA and Tukey’s test; *p* < 0.05). The average values with standard errors are presented.

The effect of Pi nutrition and AMF inoculation on plant productivity is presented in Figure 1. Phenotypes of *M. lupulina* plants at tested stages of development are represented in Figure 1A (AM-P+). The fresh weight of roots was chosen as a productivity parameter, which was determined at six key stages of *M. lupulina* plant development. The results showed the inhibitory effect of phosphorus deficiency and the positive effect of mycorrhization on root growth (Figure 1B).

The mycorrhizal growth response (MGR, symbiotic efficiency of AM), calculated on the weight of roots (Figure 1C), was reliable under conditions of phosphorus deficiency (P-) at all stages, excluding the 2nd leaf stage (2L). MGR under the condition of phosphorus treatment (P+) was significant only at the mature fruit (MF) stage, while under P- conditions it showed significance at the stage of lateral branching initiation (BI) (Figure 1C).

The values of mycorrhizal intensity in root (*M*) increased throughout the development of the host plant under P- conditions (Figure 1D). The most intense differences in the mycorrhization parameters were revealed in the AM+P+ and AM+P- variants at the third leaf (3L) and BI stages, which can be attributed to the fact that by this time AM contributed to a significant adaptation of plants to phosphorus limitation in the soil (it can be seen that the highest MGR value in the AM+P- group (variant) was observed at the BI stage; Figure 1).

### 2.2. General Characteristics of Metabolic Profiles

**Figure 2:**
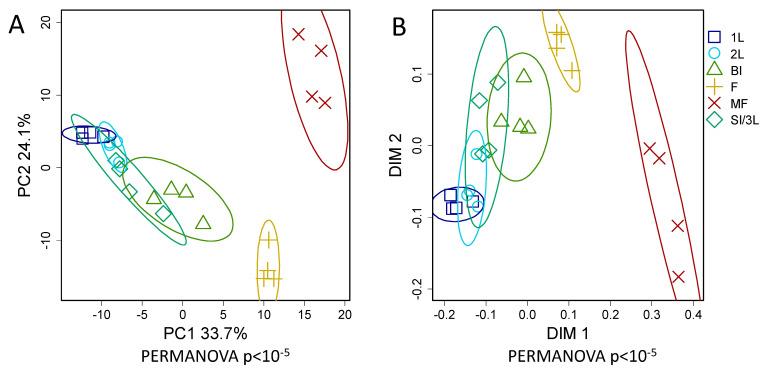
Ordination of metabolite profiles of *M. lupulina* roots at different development stages. PCA score plot (**A**). Multidimensional scaling using Spearman’s distance (1 − *r*) (**B**). The points correspond to the profiles of metabolites; % (“24.1%”and “33.7%”) is the proportion of variance associated with the principal components (PC); ellipses are 90% confidence intervals.

**Figure 3:**
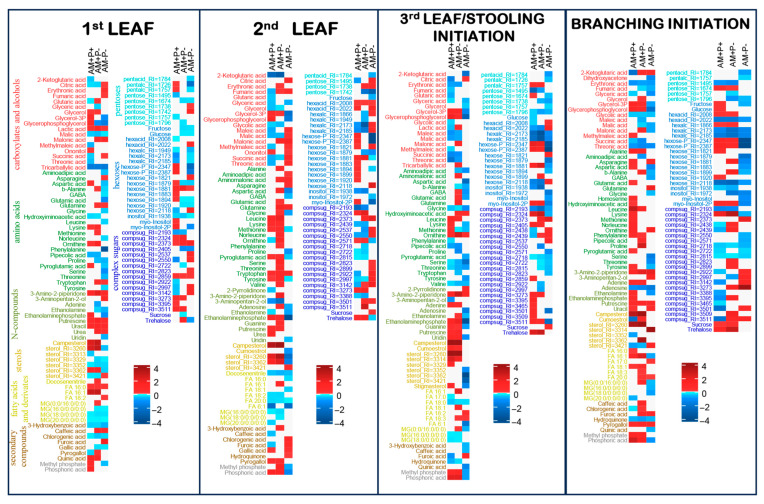
Differential accumulation of metabolites (content relative to AM-P+) at different vegetative stages of *M. lupulina* development. Heat map of the difference between the average values (normalized and logarithmic) of the metabolites content in the roots of plants in AM+P+ (inoculated with AMF and with phosphorus treatment), in AM+P- (inoculated with AMF and without phosphorus treatment), in AM-P- (without AMF inoculation and without phosphorus treatment) and the control variant AM-P+ (without AMF inoculation with phosphorus treatment). The differentially accumulated metabolites were selected according to VIP > 1 predictive components of the OPLS_DA models. The red color corresponds to a higher level relative to AM-P+. Abbreviations: compsug—complex sugar, molecules with carbohydrate moieties, hexacid—hexonic acid, hexalc—hexose alcohol, pentacid—pentonic acid, pentalc—pentose alcohol, hexose-P—hexose phosphate, MG—monoacylglycerol, FA—fatty acid, GABA—gamma-aminobutanoic acid, RI—retention index.

**Figure 4:**
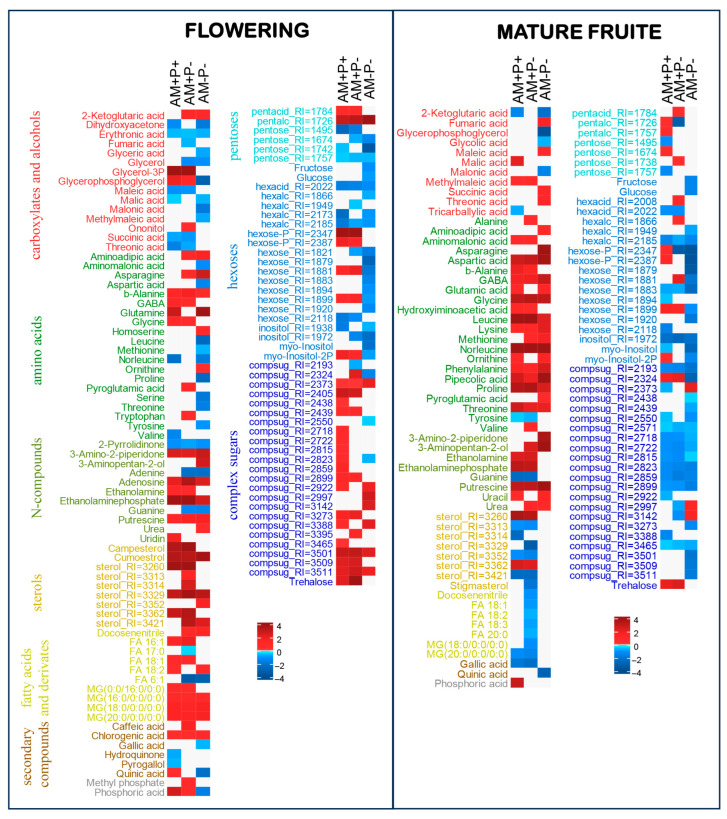
Differential accumulation of metabolites (content relative to AM-P+) at different generative stages of *M. lupulina* development. Heat map of the difference between the average values (normalized and logarithmic) of the metabolite content in the roots of plants in AM+P+ (inoculated with AMF and with phosphorus treatment), in AM+P- (inoculated with AMF and without phosphorus treatment), in AM-P- (without AMF inoculation and without phosphorus treatment) and the control variant AM-P+ (without AMF inoculation with phosphorus treatment). The differentially accumulated metabolites were selected according to VIP > 1 predictive components of the OPLS_DA models. The red color corresponds to a higher level relative to AM-P+. Abbreviations: compsug—complex sugar, molecules with carbohydrate moieties, hexacid—hexonic acid, hexalc—hexose alcohol, pentacid—pentonic acid, pentalc—pentose alcohol, hexose-P—hexose phosphate, MG—monoacylglycerol, FA—fatty acid, GABA—gamma-aminobutanoic acid, RI—retention index.

The resulting metabolic profile of *M. lupulina* roots included 327 metabolites (Figure 2, Figure 3 and Figure 4). Of these, 92 were identified and 59 more were annotated to a class (pentoses, hexoses, sterols, sugar acids, secondary metabolites, nitrogen containing glycosides). The metabolite pool included 26 amino acids, including 18 standard proteinogenic and 20 carboxylic acids, including intermediates of energy metabolism, 14 fatty acids and their derivatives, as well as nitrogenous bases, sterols, aromatic compounds and polyols. Sugars and their derivatives were most widely represented in the obtained profiles (58), including pentoses, hexoses and complex sugars.

### 2.3. Changes in the Root Metabolome During Plant Development

In order to identify and visualize the similarities and differences of the metabolomes, the profiles of plant metabolites were presented in spaces of smaller dimension obtained using principal component analysis (PCA) and multidimensional scaling (MDS). In Figure 2, the AM-P+ metabolic profiles are presented in the score plot of the first two PCs, as well as the first two dimensions obtained by the MDS method, conducted at Pearson distances (1 − *r*). The samples were grouped according to the development stages. Along PC1 (as well as DIM1), which explains 33.7% of the variance, the early stages of development were separated from the later stages (flowering and MF). Along PC2 (24.1% of the variance), the samples were also arranged according to age, but only until the flowering stage. After the transition to fruiting, the vector changed to the opposite. Thus, developmental processes played a crucial role in the determination of the metabolite profile. The most significant changes were revealed during the transition to flowering and fruiting (generative stages). Therefore, the results of metabolic profile analyses fully corresponded to the idea of plant development as an uneven and nonmonotonic process, regardless of how optimal the development conditions were.

Since the age of plants severely affected the profile of metabolites, the differences between mycorrhizal and nonmycorrhizal variants were examined separately at each point of development. The primary PCA revealed common patterns in the similarities of the obtained profiles (Figure S1; Table S1). At the 1L stage, the metabolic profiles were grouped according to both the status of mycorrhization and phosphorus. The effect of mycorrhization associated with PC1 was 26.0%, and phosphorus with PC2 was 19.5%. Thus, at this stage, the influence of both factors was expressed. At the 2L stage the situation changed. In the PC1 space (28.0%), there were no differences between AM+P+ and AM+P-; AM-P- was maximally distant from these variants, and AM-P+ was between them. Thus, the effects of phosphorus and mycorrhization were partly similar, and the effect of mycorrhization largely compensated for the lack of phosphorus. The differences between P+ and P- plants were associated with PC3 (12.3%). At the next stage, SI/3L distribution along PC1 was similar, but it already explained >40% of the variance. Thus, the effects associated with mycorrhization had increased. The differences between P+ and P- plants were associated with PC2 (14.3%), which was similar to the previous development stage. Further, at the BI stage, the distribution along PC1 was similar, but it already explained 29.5% of the variance. The effect of phosphorus on mycorrhization was weakly expressed. Some distance between AM+P+ and AM+P- was observed in the PC2 and PC3 spaces. At the flowering (FL) stage, the effect of phosphorus during mycorrhization was not revealed. AM-P+ plant metabolic profiles were formed along PC1 (29.5%), and AM-P- along PC2 (23.1%). A similar pattern was observed at the MF stage: also, the effect of phosphorus under mycorrhization was almost not pronounced. AM-P- plants were notable along PC1 (25.7%), and AM-P+ plants along PC2 (23.0%).

### 2.4. Metabolic Differences During Four Vegetative Stages Triggered by AM Without Pi Deficiency

The analysis of alteration in metabolic profiles was carried out on the basis of classification by the orthogonal partial least squares–discriminant analysis (OPLS-DA) method for each stage of development. It was shown that 31%/27%/45%/38% of the variance (at 1L, 2L, SI/3L and BI stages, accordingly) in the content of metabolites were associated with the predictive component, Q^2^Y_pred_ = 0.81, 0.87, 0.90 and 0.85 (*p* < 0.05), respectively. The results indicated that, during vegetation, the effect of mycorrhiza, even in the presence of phosphorus, in the formation of the metabolite profiles was significant. Among the differentially accumulated metabolites, compounds of various classes were revealed (Figure 3). At the vegetative stages the mycorrhization reduced the content of many carboxylates, including intermediates of the Krebs cycle. An accumulation of several amino acids (lysine, ornithine) and nitrogen metabolism intermediates was shown. An increase in the level of hexoses and sugar phosphates indirectly indicated an increase in the activity of the upper part of glycolysis. At the same time, a number of complex sugars were characterized by multidirectional changes. Mycorrhiza also caused an increase in the level of campesterol and C16 free fatty acids (FFAs). Only at the SI/3L stage was the effect of mycorrhization on the accumulation of FFA relatively weak. Among the secondary compounds, attention can be paid to an increase in the level of quinic acid and a decrease in cinnamic and gallic acids at the 1L stage. It turned out that mycorrhization increased the accumulation of phosphates, phosphorylated forms of glycerol and sugars even at an optimal level of phosphorus in the substrate. Complex multidirectional dynamics of sugar accumulation was noted, with a certain decrease in glucose accumulation and stimulation of trehalose at the 2L, SI/3L and BI stages. With a lower accumulation of glycosides in AM+P+, the content of sucrose and trehalose increased along with glucose, fructose and other hexoses at the BI stages. MSEA (Figure S2) showed that, in the case of optimal phosphorus supply, the development of AM symbiosis caused repression of the accumulation of metabolites associated with tricarboxylic acids (TCA) cycle, such as citrate and malate, and carboxylate metabolism (succinate, malate and glutaric acid, with the exception of *α*-ketoglutarate = 2-ketoglutaric acid, the level of which increased), moderate activation of amino acid-related pathways and activation of monosaccharide metabolism.

### 2.5. Metabolic Differences During Four Vegetative Stages Triggered by AM in Case of Pi Deficiency

In the OPLS-DA model, 37%/35%/37%/40% of the variance (at 1L, 2L, SI/3L and BI stages, accordingly) in the content of metabolites was associated with the predictive component, Q^2^Y_pred_ = 0.88/0.83/0.88/0.92 (*p* < 0.05), respectively. The analysis of differentially accumulated metabolites (Figure 3) revealed a decrease in the level of TCA intermediates, especially malate in AM+P-. Among carboxylates, an increase in the accumulation of malonate in AM+P- could be distinguished. The mycorrhization under phosphorus deficiency at the BI stage contributed to suppressing the accumulation of carboxylates (Figure 3), in particular citrate and malate. The data obtained might indicate the repression of the decarboxylation reaction at the BI stage. Applying the identified pool of metabolites, the changes in the intensity of various metabolic pathways were predicted using metabolite set enrichment analysis (MSEA) based on loadings from OPLS-DA classification at different vegetative stages of *M. lupulina* development (Figure S2). The amino acids with an increased content in the AM+P- at the 1L and 2L stages included glutamine, methionine and tryptophan, consistent with the accumulation of intermediates of amino acid metabolisms according to MSEA (Figure S2). Among the non-proteinogenic amino acids, the accumulation of *β*-alanine and ornithine could be noted. Other intermediates of nitrogen metabolism, such as putrescine, also had a higher content. At the SI/3L stage the accumulation of key amino acids such as glutamine, glutamate and GABA was greater. Only at the 1L stage the accumulation of uracil and the repression of adenine accumulation occurred, which might be related to the activity of the pyrimidine base metabolism indicated by MSEA. Differences in the patterns of monosaccharide content were few and almost always associated with the suppression of their accumulation, including glucose and fructose. The level of sucrose, as well as a number of other complex sugars (for example, trehalose), was also higher in AM+P-. Significant alterations occurred in the composition of lipophilic compounds. The level of monoacylglycerols (MGs) was significantly reduced, especially at the 1L and 2L stages. The accumulation of sterols decreased more often than it increased at the 1L stage, but then the content of sterols increased at the 2L, SI/3L and BI stages. A greater accumulation of campesterol could be noted. Among the FFAs, an increase in the level of 16:1 at vegetative stages could be distinguished, with a decrease in the levels of several other FFAs. Sugars showed multidirectional patterns of differences. The level of trehalose was increased. The fructose and glucose levels were greater at the 1L stage, but there were no differences for the compounds at the 2L, SI/3L and BI stages. Secondary compounds were also characterized by multidirectional changes. There was an increase in the level of pyrogallol and caffeic acids. Interestingly, there was no difference in the levels of methyl phosphate and phosphoric acid at the 1L stage, indicating that mycorrhiza at this stage coped well with the supply of phosphorus to the plant. In AM+P-, only the level of glycerophosphoglycerol was reduced. But then the levels of methyl phosphate and phosphoric acid increased at the 2L, SI/3L and BI stages.

### 2.6. Metabolic Differences During Four Vegetative Stages Triggered by Pi Deficiency Without AM

In the OPLS-DA model, 41%/33%/42%/33% of the variance (at 1L, 2L, SI/3L and BI stages, accordingly) in the content of metabolites was associated with the predictive component, Q^2^Y_pred_ = 0.91/0.83/0.92/0.81 (*p* < 0.05), respectively. At the 1L and 2L stages the TCA intermediates (citrate, fumarate and succinate) had a higher level in AM-P-, while half of the standard amino acids showed a decrease, including glutamate, proline and phenylalanine. At the same time, the levels of *α*-alanine and *γ*-aminobutyric acid (GABA) were reduced. The accumulation of tryptophan, asparagine and glutamine was higher in AM-P-, especially at the 1L and 2L stages. Other intermediates of nitrogen metabolism, such as urea, putrescine and uracil, also had a higher content but only at the 1L stage. Among monosaccharides, a decrease in glucose and fructose levels could be noted, which was probably due to a decrease in the activity of hexose metabolism at vegetative stages. However, the level of sucrose did not greatly differ. For the most part the complex sugars had shown a general trend towards higher content during phosphorus deficiency. The exception was trehalose. Attention was drawn to the lower content of several amino acids, including GABA, *β*-alanine, serine, threonine and aspartate. The MG level was reduced. The accumulation of sterols was suppressed at the 1L, 2L and SI/3L stages, but was elevated at the BI stage. FFA showed a downward trend in the first phases of AM development: higher FFA levels at the 1L stage, but lower FFA levels at the 2L stage. Together with a decrease in the level of glycerol phosphate, this indicated a repression of glycerolipid metabolism. Among the secondary compounds, an increase in the level of furoic acid was identified. The level of phosphoric acid did not differ at the 1L stage, but the level of several organic phosphates was reduced. But at the 1L, 2L and SI/3L stages, plants in AM-P- were stressed by phosphorus deficiency and the levels of methyl phosphate and phosphoric acid were decreased.

### 2.7. Comparison of the Effects of Mycorrhization and Phosphorus Treatment on Vegetative Stages of Plant Development

To compare the effects of mycorrhization in the presence and deficiency of phosphorus, the loadings of the predictive components of the corresponding OPLS-DA models were compared (Figures S3–S6). It was found that the comparison of the effects of mycorrhization under deficiency and optimal phosphorus supply confirmed their high similarity (rho = 0.58, 0.61, 0.68 and 0.52 at the 1L, 2L, SI/3L and BI stages, accordingly; *p* < 10^−16^; Figures S3–S6). As it turned out, the effects of phosphorus were quite similar (rho = 0.45, *p* < 10^−16^) at the 1L stage, but a comparison of the effects of phosphorus deficiency in the absence of mycorrhization (AM-P- vs. AM-P+) and when phosphorus was replaced by mycorrhization (AM+P- vs. AM-P+) showed that they are not similar at the 2L, SI/3L and BI stages (rho = 0.02, *p* = 0.78; rho = 0.1, *p* = 0.07; rho = 0.13, *p* = 0.02, respectively; Figures S3–S6).

### 2.8. Metabolic Differences During Two Generative Stages Triggered by AM Without Pi Deficiency

In the OPLS-DA model, 43%/29% of the variance (at FL and MF stages) in the content of metabolites was associated with the predictive component, Q^2^Y_pred_ = 0.87, 0.77 (*p* < 0.05), respectively. At the FL stage (Figure 4), mycorrhization led to the decrease in carboxylate accumulation, including malate and succinate, which was probably due to the repression of TCA cycle and the total exchange of oxocarboxylates. The elevation in amino acid content was weaker. It is worth mentioning that there was a decrease in the level of branched-chain amino acid metabolism. Together the levels of glutamine, glycine, GABA and *β*-alanine increased. The content of many nitrogen-containing compounds also increased, for example, putrescine. Complex sugars, in particular trehalose, increased their accumulation during mycorrhization. At the same time, the level of sucrose did not differ, neither did glucose and fructose. The remaining monosaccharides showed multidirectional trends. These included the repression of hexose metabolism. It is also necessary to note the trends towards an increase in the content of sterols, acylglycerols and FFA. Mycorrhization led to an increase in phosphate pools.

At the MF stage (Figure 4), the effects of mycorrhization on the accumulation of carboxylates significantly weakened; malate accumulation and a decrease in *α*-ketoglutarate were observed. The level of amino acids sharply increased, which was probably due to the repression of protein synthesis (with the exception of a decrease in tyrosine levels), and perhaps with increased proteolysis. There was no effect on the FFA level, but some differences in the accumulation of sterols were observed. The content of complex sugars decreased, but the level of trehalose was higher and the level of sucrose did not differ from its level in AM-P+ plants. The accumulation of phosphates was noted during mycorrhization. The changes in the intensity of various metabolic pathways were predicted using MSEA based on loadings from OPLS-DA classification at different generative stages of *M. lupulina* development (Figure S7). According to MSEA, at the MF stage the mycorrhization in the AM+P+ led to the upregulation of a number of amino acids, the negative effect of AM on the TCA cycle decreased and the regulation of sugar accumulation was shifted to a negative one.

### 2.9. Metabolic Differences During Two Generative Stages Triggered by AM in Case of Pi Deficiency

In the OPLS-DA model, 42%/31% of the variance (at FL and MF stages, accordingly) in the content of metabolites was associated with the predictive component, Q^2^Y_pred_ = 0.84 and 0.75 (*p* < 0.05), respectively. At the FL stage (Figure 4 and Figure S7) in the absence of a phosphorus supply, *α*-ketoglutarate was characterized by a higher level, but the accumulation of other TCA participants (fumarate and succinate) was suppressed. On the contrary, several amino acids and other nitrogen-containing compounds accumulated, for example, tryptophan, GABA, *β*-alanine and putrescine. Sugars showed various patterns, including some repression of hexose metabolism. There was a greater accumulation of sterols and acylglycerols, as well as monounsaturated C16 and C18 FFA. As in the earlier stages, mycorrhization contributed to the accumulation of phosphates.

At the MF stage (Figure 4 and Figure S7), the mycorrhization under the absence of phosphorus application stopped having an effect on the accumulation of carboxylates, while amino acids accumulated in greater quantities, with the exception of tyrosine. The level of complex sugars was reduced. The level of sterols decreased more often, including stigmasterol. Unlike the previous stages, mycorrhization under phosphorus deficiency did not contribute to the increase in content of phosphorus-containing compounds.

### 2.10. Metabolic Differences During Two Generative Stages Triggered by Pi Deficiency Without AM

In the OPLS-DA model, 47%/39% of the variance (at FL and MF stages, accordingly) in the content of metabolites was associated with the predictive component, Q^2^Y_pred_ = 0.89 and 0.82 (*p* < 0.05), respectively. At the FL stage (Figure 4 and Figure S7), the phosphorus deficiency led to an increase in the level of *α*-ketoglutarate, and a decrease in the level of fumarate, malate and some other carboxylates. Mostly, the amino acid levels were reduced. But the content of glutamine, alanine and a small number of other nitrogen-containing compounds, including ornithine and putrescine, increased. Along with this, there was a pronounced repression of the metabolic pathways of monosaccharides. Among related lipophilic compounds, an increase in the accumulation of coumestrol, acylglycerols and linoleic acid can be distinguished. It was noted that AM-P- plants contained less phosphate and phosphorylated compounds.

At the MF stage (Figure 4 and Figure S7), the phosphorus deficiency in the absence of mycorrhiza was associated with a large accumulation of fumarate and succinate, with a decrease in *α*-ketoglutarate. The accumulation of amino acids and nitrogen-containing compounds was relatively high. It is noteworthy that there was no effect on lipid metabolism. The accumulation of sugars was suppressed, and the phosphorus deficiency no longer led to a smaller accumulation of phosphorus.

### 2.11. Comparison of the Effects of Mycorrhization and Phosphorus Treatment on Generative Stages of Plant Development

At the FL and MF stages (Figures S8 and S9), the comparison of the effects of mycorrhization under deficiency and optimal phosphorus supply confirmed their very high similarity of metabolomic profiles (rho = 0.82 and 0.76, respectively; *p* < 10^−16^). The comparison of phosphorus deficiency in the absence of mycorrhization (AM-P- vs. AM-P+) and in its presence (AM+P- vs. AM-P+) showed that the effects were significantly and fairly similar (rho = 0.55, *p* < 10^−16^; rho = 0.38, *p* < 10^−12^; Figures S8 and S9, accordingly), which differed from the previous vegetative stages.

Unlike the previous stage, AM-P- plants were characterized by the repression of carbohydrate metabolism at the MF stage. The distribution of amino acids showed multidirectional trends. Thus, GABA, asparagine, phenylalanine and lysine showed high levels in AM-P-, and *β*-alanine and tryptophan in AM+P+ plants. AM+P+ plants contained more Pi, phosphorylated glycerol and sugars. It is also necessary to note the increase in the accumulation of succinate and some other carboxylates, with a lack of phosphorus and the absence of mycorrhization.

### 2.12. Metabolite Correlation Maps

**Figure 5:**
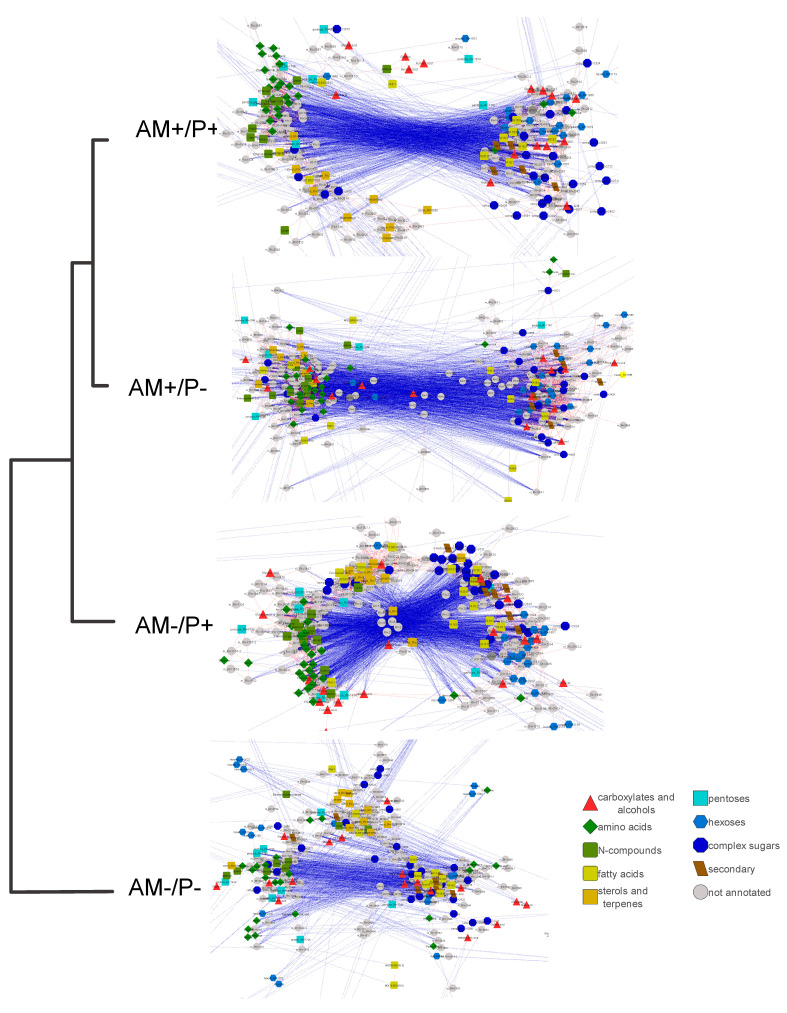
The effect of mycorrhization and phosphorus on the metabolite content correlations. Mapping of metabolites by strong (*r* > 0.90) Pearson’s correlations of average content at each point of plant development. Graphs were built in Cytoscape with “prefuse force-directed” layout. The nodes correspond to the metabolites; the shapes and colors reflect the chemical class of the compound. The edges correspond to correlations (blue—negative correlation, red—positive correlation). Positive relationships contract the nodes. The dendrogram on the left side is a result of the clustering of variants by the Ward method using distance as the similarity of correlations for pairs of metabolites, Spearman’s distance (1 − *r*).

Figure 5 shows graphs where the nodes correspond to metabolites, and the edges correspond to a strong correlation (*r* > 0.9). The networks differed both in form and characteristics (Figure 5, Table 1). Networks demonstrated heterogeneity. In addition, there were more positive relationships in all networks than negative ones, and the ratio of the number of positive to the number of negative ones increased in the absence of mycorrhiza or phosphorus deficiency. AM- and/or P- plants tended to have networks with a smaller radius. It is also interesting that the absence of mycorrhiza or phosphorus deficiency led to an increase in the average number of neighbors, a decrease in the characteristic path length and an increase in the clustering coefficient and network density; at the same time, the combination of AM-P- led to the opposite effect. Attention should be paid to less dense, greater heterogeneity, radius and diameter of networks for AM+P+ plants. Thus the network structure differed significantly. Two large clusters were expressed in AM+ networks. Nitrogen-containing compounds and sterols were concentrated in the left cluster, while in AM+P+ this cluster consisted of two regions, “sterol” and “amino acid”. In the right cluster, mainly sugars and FFA were combined. Carboxylates were located in both clusters. Carboxylates from the initial part of the TCA (citrate, 2-ketoglyctarate) were found together with sugars and FA, and carboxylates from the final part of the TCA (malate, succinate, fumarate) were found together with amino acids. In the absence of mycorrhiza, they were more heterogeneous. On the left, the AM-P+ group had split, and sterols, amino acids and other nitrogen intermediates could be distinguished. On the right, the cluster was divided into two parts: one with a predominance of glycosides and FA, and the second with monosaccharides.

In order to determine the similarity of the structure of functional relationships, the variants were clustered according to the correlation values for all pairs of metabolites (Figure 5, dendrogram). It revealed that AM+P+ and AM+P- were the closest, which indicated a relatively low effect of phosphorus availability in the presence of mycorrhiza. The absence of mycorrhiza has a stronger effect, especially under phosphorus deficiency.

## 3. Discussion

The mechanisms of effective AM development are mainly studied under a deficiency of basic macronutrients [29,31]. Conditions of P limitation are of a special interest [20,21], since AMF are able to significantly enhance the phosphate nutrition of plants. Pi limitation causes intensive responses such as (1) reduced leaf blades size and photosynthetic activity; (2) decreased plant height and productivity; (3) weak branching; (4) delayed developmental stage [24,32,33,34]. The design of the performed experiment schedule was aimed to elucidate differences in the effect of phosphorus supply and mycorrhization. The obtained results indicated that during vegetative stages a complex metabolic response formed even if it was not accompanied by significant growth alteration (Figure 1B, Figure 3 and Figure S2). From the stage of the second leaf development up to the mature fruiting stage, black medic plants with AM, both in conditions of insufficient and optimal Pi nutrition, had a higher content of phosphates (methyl phosphate and phosphoric acid) compared with the control without AM. In the presented study, the fresh weight of *M. lupulina* roots under Pi deficiency was inhibited from the 3L stage compared with that at the optimal Pi level in the substrate (Figure 1). The variant with the addition of fertilizer without AMF (AM-P+) inoculation was chosen as a control since the low Pi level in the substrate in the variant without fertilizer (AM-P-) had an inhibitory effect (Figure 1A), and both of the variants with AM inoculation (AM+P-; AM+P+) were found mostly to stimulate the growth and development of the host plant. Inoculation triggered a complicated effect; at the branching initiation stage, both variants AM- and AM+ overgrew plants at optimal Pi. Further on, AM+P- plants almost stopped root weighting, while AM+P+ accelerated it and overcame the AM-P+ variant by 30%. Thus, the obtained phenotypes indicated the importance of the BI initiation stage and the non-additive action of Pi supply and mycorrhization.

The metabolic rearrangements that can be detected in the roots of the host plant during the symbiosis development with an effective and ineffective AMF are of particular interest [35,36,37]. Long-term studies’ data of metabolic alterations triggered by symbiosis require additional attention.

Previous experiments were performed using different symbiotic pairs, PMS, but these had a number of limitations: (1) ineffective AM symbiosis is often characterized by a low increase in productivity during mycorrhization or its absence (weakly efficient PMS “*Stevia rebaudiana* Bertoni + *R. irregularis*” at 69–123 days after inoculation (dai) with different Pi treatment [22]; inefficient PMS “*Pisum sativum* (*Lathyrus oleraceus*) Lam. + *R. irregularis*” at 7–110 dai [23,38]; weakly efficient PMS “*Salvia miltiorrhiza* Bunge + *Glomus versiforme* (P.Karst.) S.M.Berch.” with different Pi treatment [30]; PMS “*M. truncatula* + *R. irregularis*” at 7–63 dai [8]; weakly efficient PMS “*Solanum lycopersicum* + *R. irregularis*” at 7–91 dai [25] and at 28–140 dai [26]); (2) only part of the plant life cycle was investigated, which made it difficult to understand whether the metabolic alterations were associated with AM symbiosis and/or related to the stage of plant development (at 120 dai [30]; at 14–24 dai [32]; at 93–134 dai with different Pi treatment [39]). Among the few efficient PMS, the following are noted: PMS “*M. lupulina* + *R. irregularis*” at 14–52 dai (our data for leaf tissues; [24]); efficient PMS “*Solidago canadensis* L. + *R. intradices*” at 45 dai [13]; efficient PMS “*Glycyrrhiza uralensis* Fisch. ex DC. + *R. irregularis*” at 90 dai with different Pi treatment [40]. However, the vast majority of studies provided under different Pi supplies did not represent detailed analyses of primary metabolites, or PMS were insufficiently effective for studying metabolic rearrangements in different Pi availability conditions. Unfortunately, data on such protracted investigations during vegetative and generative stages are absent. Therefore, our research focused on a long-lasting (from the first leaf development stage to the mature fruit) analysis of the intensity of growth and development of the host plant using the highly efficient PMS “*Medicago lupulina* + *Rhizophagus irregularis*” under conditions of limited and optimal phosphorus content suitable for plant nutrition.

### 3.1. AM Regulation of Carboxylate Accumulation

According to obtained data, the AM significantly suppressed the formation of carboxylates but stimulated the accumulation of metabolites such as glycerine, phosphates and starch in *M. lupulina* roots. At early vegetative stages (at 1L, 2L and SI/3L) the negative regulation of TCA biosynthesis of citrate, *α*-ketoglutarate, succinate and fumarate was observed in AM+P+ during mycorrhization, while citrate, succinate and fumarate biosynthesis was positively regulated in AM-P-. At the following BI stage, the TCA content (only *α*-ketoglutarate and succinate) was still low in AM+P+. But *α*-ketoglutarate was upregulated in AM-P-. During the transition to the FL and MF stages, the TCA content (succinate or *α*-ketoglutarate) was lower in AM+P+ during mycorrhization, and higher in the AM-P- variant. Analysis of the metabolic processes (Figure S10) indicated that, in the vegetative stages (1L, 2L, 3L), a clear positive regulation of glycolysis with a negative regulation of the TCA cycle was observed. It was concluded that BI was the key transitional stage of the host plant development. This triggered significant metabolic rearrangements, such as both glycolysis and the TCA cycle, and a number of other metabolic processes (Figure S10).

Such intensive variability in carboxylate accumulation mirrored different metabolic processes during host plant development and shifting AM establishment and Pi deficiency. Root exudates are an important source of organic carbon in the soil. Up to 50% of the total photosynthetic production of plants can be assimilated by the rhizosphere [41]. Moreover, nutrient uptake is one of the important indicators of root activity that is closely related to root exudates and the intensity of that depends on AM development. A significant portion of root exudates consists of high molecular weight compounds such as proteins and polysaccharides, and diverse low molecular weight metabolites. The latter include a wide range of primary (amino acids, sugars, carboxylates) and secondary (flavonoids, coumarins) metabolites. The carboxylates of root exudates are well-known chelating substances that dissolve phosphorus for plant assimilation [42]. Organic acids (including TCA) make up a significant part of exudates. It can be assumed that the accumulation of this set of primary metabolism compounds varies significantly during mycorrhization.

### 3.2. AM Upregulates Protein Biosynthesis

Analysis of key metabolic alterations in AM+P+ and AM+P- vs. AM-P+ plants (Figure S10) showed that AM plants were characterized by positive regulation of the intensification of amino acid metabolism related to protein biosynthesis: aminoacyl-tRNA biosynthesis, cofactor biosynthesis, biosynthesis of proteinogenic amino acids (valine, leucine, isoleucine, arginine, phenylalanine, tyrosine, tryptophan), metabolism of alanine, aspartate and glutamate were enhanced. Special attention can be directed to the BI stage, especially in AM+P+ variant. Such coordinated strengthening was determined also at the final FL stage. Under Pi deficiency conditions (plants AM+P-), mycorrhization led to an increase in the biosynthesis of proteinogenic amino acids in earlier vegetative stages, beginning already from the 1L and 2L stages. Surprisingly, at the SI/3L stage in both variants (AM+P+ and AM+P-), most processes associated with protein biosynthesis were downregulated. Under stressful AM-P- conditions, all stages, except MF, demonstrated a limitation of protein biosynthesis processes. Thus, the final generative stage (MF stage) was commonly characterized by the accumulation of amino acids, and, consequently, greater physiological activity in plants. The intensification of processes related to protein accumulation, including the accumulation of amino acids, is associated with the fruiting stage (and may indicate a fast aging of AM-P+ plants). According to the literature data, in the PMS “*Anchusa officinalis* L. + *R. irregularis*”, mycorrhization also led to an increased content of aspartic and glutamic acids in the roots [43]. The effective PMS “*Allium cepa* L. + *Funneliformis mosseae* + *F. constrictum* (Trappe) C.Walker & A.Schüßler + *Gigaspora margarita* (W.N.) Becker & (I.R.) Hall + *R. irregularis*” showed an increased content of the following amino acids in shoots: phenylalanine, isoleucine, leucine, histidine, lysine, methionine, threonine and valine, as well as glycine, arginine, aspartic acid, serine, glutamic acid, cysteine, alanine, tyrosine and proline [44]. In the PMS “*Solanum lycopersicum* + *R. irregularis*/*F. mosseae*” the content of asparagine, glutamic, aspartic and pyroglutamic acids in the roots was higher during mycorrhization, but these plants were characterized by lower levels of phenylalanine, tyrosine, tryptophan, alanine and leucine [21]. Thus, the effect of the AMF on the amino acid content strongly depends on the type of host plant and its cycle of development.

### 3.3. AM Upregulates the Biosynthesis of Carbohydrates

Trehalose is a disaccharide known for its role in the maintenance of osmotic balance under different stress factors such as salinity and desiccation in different organisms [45]. According to our data, the content of trehalose was significantly higher at all tested developmental stages (vegetative and generative stages) in the roots of AM+P- plants. A similar effect (besides 1L stage) was also determined in the roots of AM+P+ vs. AM-P+ plants. In plants the level of this metabolite is relatively low. The revealed accumulation in black medic roots might be associated with the predominantly fungal origin of trehalose synthesized by the AMF in the mycorrhizal roots [46]. A clear indication appeared that host plant carbohydrates were also subjected to changes. In AM+P+ vs. AM-P+ plants, the vegetative stages (1L-2L stages) were characterized by an intensification of galactose, fructose, mannose, sucrose and starch metabolism. Further on, the opposite tendency was revealed. It is noteworthy that the addition of phosphorus fertilizer (AM-P+) led to a more active metabolism of galactose, fructose, mannose and sucrose than mycorrhization (AM+P-). Hexose_RI = 1881, compsug_RI = 3273 and trehalose (Figure S10) could act as marker metabolites of carbohydrate metabolism for the development of efficient AM symbiosis with positive regulation. A similar AM-induced enhancement of protein and carbohydrate biosynthesis was obtained in studies performed with other plants: *M. truncatula* [18], *Solanum lycopersicum* [47], *Sorghum caudatum* and *S. bicolor* [35]. In the PMS “*M. truncatula* + *R. irregularis*”, an increased level of amino acids (aspartic and glutamic acid and lysine) was observed in AM plants, with unreliable differences in sugar levels both under conditions of deficiency and with a sufficient Pi level [18]. In the PMS “*Solanum lycopersicum* + *R. irregularis*” and “*Solanum lycopersicum* + *F. mosseae*”, the upregulation of amino acids (aspartic and glutamic acid and histidine) was observed [47]. On the other hand, in the inefficient PMS “*Vitis vinifera* L. + *R. irregularis*”, mycorrhization led to a limitation in sugars [17]. Very interesting data were obtained from the comparative analysis of highly efficient PMS “*Sorgum caudatum* + *R. irregularis*” and “*S. bicolor* + *R. irregularis*” and inefficient PMS “*S. caudatum* + *Gigaspora gigantea* (T.H.Nicolson & Gerd.) Gerd. & Trappe” and “*S. bicolor* + *G. gigantea*” [35]. Under Pi deficiency at 30 dai, highly efficient PMS showed the upregulation of carbohydrates (galactose, glucose, fructose, sucrose and trehalose), as well as TCA (citrate, succinate, fumarate and malate), with a negative regulation of GABA and a number of proteinogenic amino acids (serine, lysine, threonine, tyrosine, aspartic acid, glycine, glutamine, valine, leucine and isoleucine) [35]. Inefficient PMS “*Sorgum caudatum* + *G. gigantea*” and “*S. bicolor* + *G. gigantea*” were characterized by a reverse regulation of all listed metabolites, except a positive regulation of sucrose [35]. The presented data are consistent with the results of our study in the PMS “*M. lupulina* + *R. irregularis*”, obtained approximately at the same time point (at 28 dai = 28 DAS) at the SI stage, characterized by a significant decrease in the upregulation of metabolism of amino acids, carbohydrates and lipids up to the negative regulation of some metabolites. This comparison indicates the importance of more long-lasting dynamic analysis of PMS “*Sorgum caudatum* + *R. irregularis*”, “*S. bicolor* + *R. irregularis*”, “*S. caudatum* + *G. gigantea*” and “*S. bicolor* + *G. gigantea*”, with the inclusion of analysis at the next developmental stage (at ~35 dai).

### 3.4. AM Effect on Energy Metabolism

During mycorrhization, an increase in glycolysis, biosynthesis of pantothenate and cofactor A was observed (in both AM+P+ and AM+P-; Figure S10). But the metabolism of pyruvate and TCA carboxylates related to energy transformation was repressed during mycorrhization in both variants. It was shown that, both at early and late developmental stages, the downregulation of the TCA cycle was observed (Figure 3, Figure 4, Figures S2 and S7). Revealed earlier [32], the inhibition of the early stages of the Krebs cycle, as well as the intensification of transamination reactions, might result in the rapid depletion of the ketoacids pool due to activation of mitochondrial and plastid metabolism during mycorrhization [18,48], and a negative effect of AM symbiosis on central catabolism. The presented data showed that the content of citric acid (citrate), *α*-ketoglutarate, glutamate, succinate and fumarate at the vegetative stages (1L, 2L, 3L/SI) was reduced most of all in AM+P+ plants. In AM+P- plants, the content also declined at the key developmental stages, except for *α*-ketoglutarate. The opposite trend was observed at the BI and FL stages. The negative regulation of the TCA cycle was previously shown in the roots in the efficient “*M. truncatula* + *R. irregularis*” PMS (primarily for fumarate and cisaconate) at 42 dai [18]. So the content of citrate, succinate, fumarate and malate in inefficient dicot PMS “*M. truncatula* + *R. irregularis*” was reduced [19]. In another dicot, in efficient PMS “*Ocimum tenuiflorum* L. + *Rhizophagus intraradices*” at 120 DAS (at the MF stage), the AM induced glycolysis and repressed the Krebs cycle [49]. Metabolome profiling of AMF-treated *O. tenuiflorum* provided insights into deviations in the allocation of carbon compounds to secondary metabolism. Positive AM regulation of the TCA cycle in *Plantago major* L., *P. lanceolata* L., *M. truncatula*, *Veronica chamaedrys* L. and *Pisum sativum*, forming an inefficient symbiosis with *R. irregularis*, was observed [19,23]. Negative AM regulation of the TCA cycle was also found under conditions of deficiency and a sufficient level of Pi in the substrate in the “*M. truncatula* + *R. irregularis*” PMS [18]. Similar data (reduced levels of citric acid, succinic acid and malic acid) were previously also obtained in leaves in the “*Lotus japonicus* (Regel) K.Larsen + *F. mosseae*” PMS at 70–84 dai under Pi deficiency conditions [50]. The opposite reaction (positive regulation of the TCA cycle) was determined in the roots of monocot cereal (Poaceae) in the “*Poa annua* L. + *R. irregularis*” and “*Hordeum vulgare* L. + *Glomus* sp.” PMS [51], which may be due to differences in the metabolism of dicotyledonous and monocotyledonous plants. Thus, monocots, for example cereals (Poaceae), are characterized by a lower AM colonization of roots compared with dicotyledonous species, which is consistent with the results of other studies [52]. Differences in colonization levels may reflect opposite strategies for obtaining nutrients. For example, according to [53], monocotyledonous species allocate more resources to the development of the root system, and dicotyledonous plants, including *M. lupulina*, develop more symbiotic structures. Thus, under conditions of high Pi levels, in cereal PMS “*Triticum durum* + a mixture of strains of nine species of AMF (including *R. irregularis*)” with a weak response to mycorrhization [29], it was shown that inoculation reduced the concentration of most compounds in all metabolic pathways in roots, especially amino acids and saturated FA, while the activity of amination in the roots decreased, most likely due to the transition from the biosynthesis of common amino acids to the biosynthesis of GABA [29]. In addition, positive AM regulation of the TCA cycle may play a role in the activation (for citric acid, succinate) and suppression (for cis-aconitate, *α*-ketoglutarate, fumarate) of the immune responses [54]. Thus, in the “*T. aestivum* + *R. intraradices*” PMS, an increased level of citric acid (as well as succinic acid) in the leaves was observed with the addition of As, but the level of pyruvic acid under stress was significantly lower [55]. An increased level of tricarboxylic acids (fumaric acid, malic acid) was observed during salt stress in leaves in the “*Zea mays* L. + *F. mosseae*” PMS [56]. In the AM absence under the salt effect, an increased level of succinic acid was observed in the *T. aestivum* leaves, with a reduced level of citric acid, cis-aconitate, fumaric acid and malic acid [57]. It is known that, under salt stress, all TCA enzymes are inhibited by salt, but this inhibition is overcome by increasing the activity of the GABA shunt, which provides an alternative carbon source for mitochondria, bypassing salt-sensitive enzymes, which promotes enhanced respiration [57]. TCA metabolites can play an important role in chelation of metals and nutrients, redox regulation, can bind signaling proteins, serve as precursors of phytohormones and can also be regulated by them [58]. Thus, the analysis of organic acids of the TCA should be detailed and accurate in assessing the influence of stress factors in sensitive PMS and should be evaluated depending on the plant developmental stages.

During mycorrhization, an increase in glycolysis, biosynthesis of pantothenate and cofactor A was observed (in both AM+P+ and AM+P-; Figure S10). But the metabolism of pyruvate and TCA carboxylates related to energy transformation was repressed during mycorrhization in both variants. It was shown that, both at early and late developmental stages, the downregulation of the TCA cycle was observed (Figure 3, Figure 4, Figures S2 and S7). Revealed earlier [32], the inhibition of the early stages of the Krebs cycle, as well as the intensification of transamination reactions, might result in the rapid depletion of the ketoacids pool due to activation of mitochondrial and plastid metabolism during mycorrhization [18,48], and a negative effect of AM symbiosis on central catabolism. The presented data showed that the content of citric acid (citrate), *α*-ketoglutarate, glutamate, succinate and fumarate at the vegetative stages (1L, 2L, 3L/SI) was reduced most of all in AM+P+ plants. In AM+P- plants, the content also declined at the key developmental stages, except for *α*-ketoglutarate. The opposite trend was observed at the BI and FL stages. The negative regulation of the TCA cycle was previously shown in the roots in the efficient “*M. truncatula* + *R. irregularis*” PMS (primarily for fumarate and cisaconate) at 42 dai [18]. So the content of citrate, succinate, fumarate and malate in inefficient dicot PMS “*M. truncatula* + *R. irregularis*” was reduced [19]. In another dicot, in efficient PMS “*Ocimum tenuiflorum* L. + *Rhizophagus intraradices*” at 120 DAS (at the MF stage), the AM induced glycolysis and repressed the Krebs cycle [49]. Metabolome profiling of AMF-treated *O. tenuiflorum* provided insights into deviations in the allocation of carbon compounds to secondary metabolism. Positive AM regulation of the TCA cycle in *Plantago major* L., *P. lanceolata* L., *M. truncatula*, *Veronica chamaedrys* L. and *Pisum sativum*, forming an inefficient symbiosis with *R. irregularis*, was observed [19,23]. Negative AM regulation of the TCA cycle was also found under conditions of deficiency and a sufficient level of Pi in the substrate in the “*M. truncatula* + *R. irregularis*” PMS [18]. Similar data (reduced levels of citric acid, succinic acid and malic acid) were previously also obtained in leaves in the “*Lotus japonicus* (Regel) K.Larsen + *F. mosseae*” PMS at 70–84 dai under Pi deficiency conditions [50]. The opposite reaction (positive regulation of the TCA cycle) was determined in the roots of monocot cereal (Poaceae) in the “*Poa annua* L. + *R. irregularis*” and “*Hordeum vulgare* L. + *Glomus* sp.” PMS [51], which may be due to differences in the metabolism of dicotyledonous and monocotyledonous plants. Thus, monocots, for example cereals (Poaceae), are characterized by a lower AM colonization of roots compared with dicotyledonous species, which is consistent with the results of other studies [52]. Differences in colonization levels may reflect opposite strategies for obtaining nutrients. For example, according to [53], monocotyledonous species allocate more resources to the development of the root system, and dicotyledonous plants, including *M. lupulina*, develop more symbiotic structures. Thus, under conditions of high Pi levels, in cereal PMS “*Triticum durum* + a mixture of strains of nine species of AMF (including *R. irregularis*)” with a weak response to mycorrhization [29], it was shown that inoculation reduced the concentration of most compounds in all metabolic pathways in roots, especially amino acids and saturated FA, while the activity of amination in the roots decreased, most likely due to the transition from the biosynthesis of common amino acids to the biosynthesis of GABA [29]. In addition, positive AM regulation of the TCA cycle may play a role in the activation (for citric acid, succinate) and suppression (for cis-aconitate, *α*-ketoglutarate, fumarate) of the immune responses [54]. Thus, in the “*T. aestivum* + *R. intraradices*” PMS, an increased level of citric acid (as well as succinic acid) in the leaves was observed with the addition of As, but the level of pyruvic acid under stress was significantly lower [55]. An increased level of tricarboxylic acids (fumaric acid, malic acid) was observed during salt stress in leaves in the “*Zea mays* L. + *F. mosseae*” PMS [56]. In the AM absence under the salt effect, an increased level of succinic acid was observed in the *T. aestivum* leaves, with a reduced level of citric acid, cis-aconitate, fumaric acid and malic acid [57]. It is known that, under salt stress, all TCA enzymes are inhibited by salt, but this inhibition is overcome by increasing the activity of the GABA shunt, which provides an alternative carbon source for mitochondria, bypassing salt-sensitive enzymes, which promotes enhanced respiration [57]. TCA metabolites can play an important role in chelation of metals and nutrients, redox regulation, can bind signaling proteins, serve as precursors of phytohormones and can also be regulated by them [58]. Thus, the analysis of organic acids of the TCA should be detailed and accurate in assessing the influence of stress factors in sensitive PMS and should be evaluated depending on the plant developmental stages.

Interestingly, AMF also affected both the glyoxylate pathway and the GABA shunt, which is a bypass branch of the TCA cycle. Increasing the enzymatic activity of the GABA shunt is one of the strategies of AM plants for the predominant synthesis of GABA, rather than proline, from a common precursor, glutamate [59]. GABA is a non-proteinogenic amino acid with wide physiological importance. The elevation of GABA levels well coincides with plant resistance to different biotic and abiotic stresses [60]. Our detailed analysis of the developmental stages of *M. lupulina* showed that a significant increase in GABA content in the AM+P+ and AM+P- vs. AM-P+ in the roots of *M. lupulina* was observed only at the generative (FL and MF) but not at the vegetative stages (Figure 3, Figure 4, Figures S2 and S7).

Of interest is that, in AM+P+ and AM-P- variants, the negative regulation of furoic acid biosynthesis was observed at the vegetative stages, with a positive effect on AM-P- (Figure 3). But at the generative stages (FL and MF), there were no differences in the content of furoic acid (Figure 4). Furoic acid is known for its strong antibacterial and nematicidal effects [61], which is probably a cause for its biosynthesis suppression during mycorrhization. But, what is even more important in relation to energy metabolism, 2-furoic acid lowers citrate lyase, acetyl CoA synthetase activity and even triglyceride levels in animals.

Phosphate metabolism is closely linked to energy and sugar metabolism. In our experiments, the phosphates (methyl phosphate and phosphoric acid) could act as marker metabolites of energy metabolism for the development of efficient AM symbiosis with positive regulation, and succinate and, to a lesser extent, citrate, with negative regulation (Figure S10).

### 3.5. AM Upregulates the Lipid Biosynthesis

An analysis of the literature data showed some features of the AM effect on carbohydrate and amino acid metabolism in the roots of the host plant with a deficiency and a sufficient Pi level, but so far the features of the dynamics of lipid metabolism and energy metabolism have not been fully disclosed regarding efficient AM symbiosis development.

The mycorrhization of *M. lupulina* with *R. irregularis* led to modulation in the accumulation of FFA and lipids. In our experiments, the metabolism of glycerolipids, glycerophospholipids and fatty acids had a predominant positive regulation during mycorrhization both under low Pi conditions and under conditions of sufficient levels (AM+P+ and AM+P-, respectively; Figure S10). The largest number of probable marker metabolites for the development of efficient AM symbiosis was revealed in the lipid metabolism group: glycerol, glycerol-3P, ethanolamine phosphate, campesterol, sterol_RI = 3260, sterol_RI = 3362 and myo-inositol-2P (Figure S10). The levels of fatty acids such as FFA 16:0, 16:1, 17:0, 18:0, 18:1, 18:2, 18:3 and 20:0 were, as a rule, higher in plants AM+P+ and AM+P-. It is important to note that, at the generative stage, the positive regulation of sterols and FFA by mycorrhization in the FL stage was replaced by an intensively negative regulation in the MF stage. In general, in AM+P+ vs. AM-P+ plants, AM caused a greater accumulation of phosphorylated forms of glycerol and phosphates, as well as sterols. AM+P- vs. AM-P+ showed a higher relative phosphate level of both inorganic and organic phosphorus (for example, in 2L stage). In the inefficient PMS “*Vitis vinifera* + *R. irregularis*”, mycorrhization also led to the significant positive regulation of certain fatty acids (C16:1, C18:2, C18:3, C20:4, C20:5) [17]. Lipids are known to stimulate the growth and branching of the mycelium of the AMF [62]. Actually, all fatty acids contained in fungal lipids are probably derived from host plants (since AMF lack de novo cytosolic fatty acid synthase genes [63]). Lipids are the main source of organic carbon delivered to the fungus [64]. Some lipids act as triggers regulating the development of AMF. However, the mechanisms by which AMF import lipids are still unknown [62]. Thus, the AMF can import both the acyl and glycerol groups of 2-monoacylglycerols exported from plants [65], but it remains unclear whether 2-monoacylglycerols can be directly imported or whether they are hydrolyzed in the periarbuscular space to free fatty acids and glycerol or converted to other derivates before uptake. Furthermore, fatty acids (C16:1, C15:0, C14:0) are able to significantly stimulate the development of fungal spores [66,67]. In addition, lipid biosynthesis enzymes (AM specific) such as FatM and RAM2 are necessary for effective AM symbiosis. FatM increases the outflow of 16:0 fatty acids from plastids for subsequent RAM2 use to produce 16:0 *β*-monoacylglycerol, which is transferred to the AMF through the periarbuscular membrane [68].

### 3.6. The Effect of Mycorrhization and Phosphorus on Metabolite Interrelations

Analysis of the relationships between substances is an important aspect of systemic biological research and is used to assess how closely metabolites are related [69]. Since stimuli cause changes in the activity of transporters and enzymes binding pools of metabolites, this, in turn, will be reflected in their correlation patterns [70,71,72]. It is known that those patterns are specific to organs and tissues [73,74], to the genotype [74] and to environmental conditions [75,76,77]. The revealed alterations in metabolic pathways in PMS “*M. lupulina* + *R. irregularis*” were closely interrelated, as reflected in the intensity of correlations. The clustering of variants based on the similarity of correlations of average values (Figure 5) showed that mycorrhizal plants were the most similar to each other, and non-mycorrhizal plants were close to them under fertilizer supply conditions. The most peculiar were non-mycorrhizal plants with phosphorus deficiency. Thus, mycorrhization is the most important driver determining the pattern of metabolite relationships in the context of development. The fact that plants without mycorrhiza but with sufficient phosphorus levels turned out to be closer to mycorrhizal ones indicates that the effects of mycorrhiza on metabolic connections are associated with the phosphorus supply to the plant.

The mapping of metabolites by strong correlations is often used to analyze and visualize the correlation pattern. To reveal the AM influence on the structure of interrelations between metabolite levels and dynamic changes during development, they were mapped by strong correlations of their average content at each time point. The networks differed both in appearance (Figure 5) and in traits (Table 1), which indicated significant functional differences between the variants. The networks turned out to be similar in structure to scale-free networks. This pattern is often found in the analysis of biological systems. A characteristic trait of such networks is heterogeneity [78]. In our case (Figure 5), heterogeneity manifested itself in the fact that two large clusters were expressed in AM+ networks. Nitrogen-containing compounds and sterols were concentrated in one of the clusters; in the case of AM+P+, this cluster formed two regions. The other cluster contained mainly sugars and FA. The TCA cycle intermediates did not form a single group. Carboxylates from the initial part of the TCA cycle turned out to be together with sugars and FA, and carboxylates from the final part of the TCA cycle turned out to be together with amino acids. The formation of clusters in the correlation networks by compounds that were related metabolically was observed earlier [23,74,76,77]. Under AM-, networks became less homogeneous; in particular, the group with sterols separated. In AM-P+ plants in the right cluster, one region was distinguished, with a predominance of glycosides and FA, and the second with monosaccharides.

Interestingly, the AM+P- and AM-P+ networks differed in the largest number of edges. For AM+P+, the number was less by a quarter, and for AM-P-, it was almost half as much. Correlations are supposed to result from a combination of all reactions and regulatory processes [70,71,72]. This is possibly due to stricter regulations under only mycorrhization or only Pi deficiency. A characteristic feature of the networks was the predominance of positive correlations (Table 1). Positive correlations are observed in various biological systems, including roots and leaves of *Arabidopsis thaliana* (L.) Heynh. [73], human tumor cell cultures [77] and microalgae cultures [79]. The reason for the high positive correlation may be, on the one hand, proximity to equilibrium and a high level of metabolic flow [70,80]; on the other hand, negative correlations between metabolite levels are determined by the energy conservation law [71]. The ratio of the number of positive to the number of negative correlations increased under AM absence and/or under phosphorus deficiency. The lowest ratio of positive and negative correlations is typical for AM+P+ plants. This may be the result of an intense metabolism between the plant and the fungus. It is known that, in exchange for macronutrients (mainly phosphates), AM consumes up to 20% of photoassimilated carbon [4]. With a more intense outflow of photoassimilates, there may be some metabolites deficiency, which determines a large proportion of negative correlations.

One obvious reason for the correlation is the metabolic relationship between the metabolites. On the other hand, plant cells are highly compartmentalized spaces in which enzymes and transporters are subject to sophisticated regulatory complexes. This complicates the picture of correlation, making metabolite interactions indirect and complicated. AM symbiosis triggers metabolic reprogramming, interfering with ontogenetic and adaptive strategies. Furthermore, AM symbiosis increases the complexity of compartmentalization, and the molecular interplay between the plant and fungi increases metabolic sophistication. Consequently, AM+P+ plants have less dense correlation networks, characterized by a greater heterogeneity, radius and diameter.

### 3.7. Key Metabolic Rearrangements During the Host Plant Development

A significant effect of phosphorus fertilizer and mycorrhization on changes in the metabolic profiles of *M. lupulina* roots during development was revealed. At the first vegetative stage (1L; Figure S3), the effects were similar, but at the further vegetative stages (2L, SI/3L, BI; Figures S4–S6), the effect of these factors was completely non-additive and multidirectional. On the other hand, the generative stages (FL and MF; Figures S8 and S9) were characterized by a high similarity of metabolic profiles under conditions of deficiency and optimal Pi level in the substrate. Thus, mycorrhization with *R. irregularis* led to the adaptation of *M. lupulina* plants to low Pi level at the vegetative stages of host plant development (2L, SI/3L, BI).

The results of the presented study showed that the largest number of changes in the metabolic processes (Figure S10) were determined at the BI and MF stages. Other stage transitions, 1L-2L, 2L-SI and BI-FL, had ~1.5 times fewer changes in metabolic profiles on average. Metabolic alterations coincided with symbiotic efficiency being maximal under conditions of a low Pi level (AM+P-) at the branching initiation (BI) stage. But the symbiotic efficiency under phosphorus supply (AM+P+) was minimal during this stage (Figure 1C), which was shown for the first time. These data correlated with high rates of mycorrhization (*M*) at the BI stage in AM+P- plants and low values of these parameters in AM+P+ plants at the 3L/SI and BI stages (Figure 1D). It was estimated that, with the development of lateral branching (BI) in plants, multidirectional metabolic rearrangements occurred under conditions of a low and sufficient level of Pi in the substrate for plant nutrition. In particular, during the transition from the 3L/SI to the BI stage, *M. lupulina* plants under an optimal (supra-optimal) Pi level (AM+P+) showed an increase in protein biosynthesis, carbohydrate metabolism (galactose, fructose and mannose), lipid metabolism, pantothenate and CoA biosynthesis, but there was no increase in the biosynthesis of steroids and terpenoids. Under Pi deficiency but in the presence of mycorrhiza in AM+P- plants, no significant increase in the biosynthesis of proteinogenic amino acids was observed at the BI stage. On the contrary, there was an increase in starch and sucrose metabolism, with a negative regulation of galactose, fructose and mannose metabolism. And AM+P- plants at the BI stage were characterized by increased lipid metabolism while maintaining significant activity of steroid and terpenoid biosynthesis (Figure S10). Quite a number of studies are aimed at research at the late developmental stage in *Lotus japonicus* at 84 dai [50]; *Plantago lanceolata*, *P. major*, *Veronica chamaedrys*, *M. truncatula*, *Poa annua* at 62 dai [19]; *Senecio jacobaea* (*Jacobaea vulgaris*) Gaertn. at 70 dai [81]; *Vitis vinifera* at 60 dai [17]; *Withania somnifera* (L.) Dunal, *Tagetes erecta* L. at 90 dai [82]; *Glycyrrhiza glabra* L. at 180 dai [82]; *Elymus nutans* Griseb., *E. sibiricus* at 85 dai [83]; *Leymus chinensis* (Trin.) Tzvelev at 127 dai [84]. On the other hand, there are very few studies covering the effect of a wide range of stage transitions of the host plant during the AM development on the metabolome [18,22,23,24,25,26,38]. Only in the study of “*M. truncatula* + *R. irregularis*” PMS [8] it was shown that approximately at the BI stage (at 42 dai), the gene expression of both the fungal RiPT7 transporter and the plant phosphate MtPT4 transporter was highest, which was consistent with the pronounced AM efficiency. It can be concluded that the BI stage is critical for metabolic rearrangements in the development of efficient AM symbiosis. As an assumption, generative changes from the FL to the MF stage were usually associated with differences in the aging processes of the host monocarpic plants and not closely related with AM symbiosis. Only one vegetative transition, from the SI to the BI stage (the initiation of lateral branching), was associated with critical changes in AM, both under conditions of low and high Pi level.

## 4. Materials and Methods

### 4.1. Plant and Fungus Biomaterials

*Medicago lupulina* L. subsp. *vulgaris* Koch MlS-1 line, characterized by high AM symbiotic efficiency (mycorrhizal growth response (MGR)), was used under conditions of deficiency or abundance of Pi. The efficient strain RCAM00320 *Rhizophagus irregularis* (Błaszk., Wubet, Renker & Buscot) C. Walker & A. Schüßler was isolated in Laboratory No. 4 of Ecology of Symbiotic and Associative Rhizobacteria at All-Russia Research Institute for Agricultural Microbiology, ARRIAM; the strain was previously known as strain CIAM8 *Glomus intraradices* Shenck&Smith. The strain forms a highly efficient AM symbiosis with most crops [85,86,87,88] and was identified by members of the authors’ team [89]. *R. irregularis* is an obligate symbiont of plants, therefore the culture of AMF was grown in *Plectranthus australis* R. Br. (=*P. verticillatus* (L.f.) Druce) in Laboratory No. 4 at ARRIAM. The preparation of the AMF inoculant was provided according to [24]: step 1—*Plectranthus* cuttings were surface-disinfected with 0.5% sodium hypochlorite for 60 min and then thoroughly rinsed three times with sterile water for 30 min; step 2—disinfected cuttings were inoculated with two 10 mm root fragments of *Plectranthus* and were sown in the growth substrate with low Pi as described in “Experimental design and plant growth conditions”; step 3—after 180 days of cultivation under conditions described in “Experimental design and plant growth conditions” the root systems of *Plectranthus* accumulative culture were extracted from the growth substrate, cut into 10 mm pieces and then mixed; step 4—mixed fungal inoculum (consisting of ~100 vesicles of AM fungus per 1 seedling) was used to inoculate *M. lupulina* model plants in the experiment.

### 4.2. Experimental Design and Plant Growth Conditions

The micro-vegetative method provided optimal conditions for the development of AM and allowed to avoid spontaneous infection with rhizobia and other symbiotic microorganisms. The light box was previously subjected to UV sterilization; the substrate (soil–sand mixture) and instruments were sterilized by autoclaving; the seeds were scarified; the water for watering was boiled (the method was used earlier [24]). A substrate for cultivation, a mixture of air-dry soil and sand in a ratio of 2:1, was treated in an autoclave at 134 °C, 2 atm for 1 h, with repeated treatment in an autoclave after 2 days (no toxicity after treatment). Agrochemical characteristics of the soil: the sod–podzolic loam–poor soil with a very low phosphorus content—23 mg P_2_O_5_/kg and K_2_O—78 mg/kg (calculated using the Kirsanov reaction); organic matter content—3.64%; pH_KCl_—6.4 (after liming), pH_H2O_—7.3 according to [24]. To simulate sufficient phosphorus nutrition of plants, phosphorus fertilizer in the form of CaH_2_PO_4_·2H_2_O was applied into half of the pots before the experiment (AM+P+ and AM-P+ variants), according to Pryanishnikov’s protocol [90]. The final available phosphorus content in the substrate was 165 mg P_2_O_5_/kg. *M. lupulina* seeds were scarified for 5 min in concentrated H_2_SO_4_. The seeds were then stratified in Petri dishes for 1 day at +5 °C, and then germinated for 2 days at +27 °C in the dark. Seedlings of the same size were grown in a soil–sand substrate. Half of the plants at each level of Pi (deficiency and optimal phosphorus level) were inoculated with AM inoculant (*Plectranthus* roots mycorrhized with RCAM00320 strain *R. irregularis*) simultaneously with planting (AM+P- and AM+P+ variants), and the other half was treated with non-mycorrhized *Plectranthus* roots (AM-P- and AM-P+ variants). The control for assessing the effect of AM and Pi treatment on the metabolic profile of the roots was the AM-P+ variant, characterized by the absence of the influence of a stress factor—a low level of Pi in the absence of AM. The plants were grown by 2 seedlings in 1 pot filled with 210 g of soil–sand substrate. Plant watering was carried out every other day up to 0.6 of saturated water content. A light box was sterilized by ultraviolet radiation. The day/night regime was 18 h/6 h, and the air temperature was 24–26 °C. Biochemical and microscopic analyses of plants were performed in 6 stages of the host plant development: (1) at the 14th day after sowing and inoculation (DAS), the 1st true leaf development stage; (2) at the 21st DAS, the 2nd leaf development stage; (3) at the 28th DAS, the 3rd leaf development stage, the schooling initiation stage; (4) at the 35th DAS, the lateral branching initiation stage; (5) at the 42nd DAS, the flowering stage; and (6) at the 56th day, the mature fruit stage. The fresh mass of roots and aboveground parts of plants the height of the main stem of the plant were determined. For subsequent biochemical analysis, the roots of 8 plants were collected for 1 biological repeat (3 biological repeats per 1 treatment option), weighed and quickly frozen in liquid nitrogen, and then stored at a temperature of −80 °C.

### 4.3. Evaluation of Mycorrhization

The method of maceration and staining of root samples, designed by J.M. Phillips and D.S. Heyman for the estimation of AM infection in the roots of leguminous plants [91], was used for the microscopic analysis of AM development, including staining with trypan blue in the solution containing 90% lactic acid solution, glycerine, distilled water and “Trypan blue” dye in a ratio of 62 mL: 63 mL: 875 mL: 0.3 g, respectively. For AM analysis, the roots were dried at room temperature, then macerated and stained with trypan blue.

Sample preparation was performed on a Stemi 2000 stereo microscope (Zeiss, Oberkohen, Germany). An Olympus CX43 microscope (OLYMPUS, Tokyo, Japan; specifications: wide-field eyepiece 10× with field of view 20; Plan Achromat PLCN10X-1-7 lens with magnification 10×, WD 10.5 mm) was used for the microscopic analysis of AM structures in a squash preparation—stained, macerated and cut (1 cm in length) roots of *M. lupulina*. At least eight biological replicates were performed for each studied variant.

Mycorrhization index, *M* (the intensity of root mycorrhization), was calculated according to [92]. Microscopic analysis of the AM development was carried out using a computer program for calculating the mycorrhization indicators of plant roots, developed by Yurkov et al. [93].

### 4.4. Evaluation of Mycorrhizal Growth Response—AM Symbiotic Efficiency

The mycorrhizal growth response (MGR, symbiotic efficiency of AM) was calculated as an increase in the raw weight of shoots and roots the height of the plant stem, using the well-known formula of Odum:

MGR = ((AM+) − (AM-)) × 100%/(AM-), (1)

where (AM+) is the value of the productivity parameter in mycorrhizal plants; (AM-) is the value of the productivity parameter in plants without AM. MGR is evaluated independently for low-level Pi (P-) variants and under the conditions of phosphorus fertilizer (P+) application.

### 4.5. GC-MS Analysis

Root samples, weighing 100 mg, were collected at 6 different stages of cultivation and immediately frozen in liquid nitrogen. The plant material was crushed using a mill (MM 400, Retsch, Haan, Germany). The metabolites were then extracted using 2 mL of an extraction mixture—methanol, chloroform and water (5:2:1)—with shaking 900 rpm at 4 °C on a thermo shaker (TS-100C, BioSan, Riga, Latvia). Tissue debris was removed by centrifugation at 12,000× *g* for 10 min at 4 °C and the resulting supernatant was collected and evaporated in a vacuum evaporator (CentriVap, Labconco, Kansas City, MO, USA). Dried samples were dissolved and derivatized in pyridine at a ratio of BSTFA:TMCS 99:1 (Sigma-Aldrich, St. Louis, MO, USA) at 90 °C for 20 min. An internal standard of triclosan (normal hydrocarbon) was added.

Samples were analyzed with an Agilent 6850 chromatograph (under the control of MassHunter software v. 10.1, Agilent Technologies, Santa Clara, CA, USA) equipped with an Rxi-5Sil (Restek) capillary column and coupled with an Agilent 5975 quadrupole mass selective (Agilent Technologies, Santa Clara, CA, USA). The flow rate of the helium was 1 mL/min. The inlet was operated in splitless mode at 250 °C. Column temperature regime: initial—70 °C, final—320 °C, rate—6 °C per min. Electron impact ionization was performed at 70 V and an ion source temperature of 230 °C. The analysis of the GC-MS data was processed using the PARADISe software v. 6.0.1 (Department of Food Science, Faculty of Science, University of Copenhagen, Denmark [94]) coupled with NIST MS Search (National Institute of Standards and Technology (NIST), Gaithersburg, MD, USA). The AMDIS system (Automated Mass Spectral Deconvolution and Identification System, NIST, USA) was used for the deconvolution and identification of metabolites. Analytes were identified by mass-spectra and Kovats retention indices using libraries: NIST2020, Golm Metabolome Database (GMD; [95]). In addition, the “in house” library created by the laboratory of analytical phytochemistry with the financial support of the BIN RAS No. 124020100140-7 was used.

### 4.6. Statistical Analysis

The analysis of variance (ANOVA) and post hoc Tukey’s HSD test was used to assess the statistical significance (*p* < 0.05) of the differences in parameters of productivity, MGR and mycorrhization. Statistical analysis of the metabolomic data was processed using R 4.3.1 [96]. Data were normalized against the sample median. Outliers were detected and excluded on the basis of Dixon’s test in the *outliers* package v. 0.15 [97]. The data were log-transformed and standardized. If a compound was not detected in a sample but was present in the other replicates it was considered a technical error and imputed by KNN (k-nearest neighbor) with the *impute* R package v. 1.74.1 [98]. PCA (principal component analysis) was performed with *pcaMethods* v. 1.92.0 [99]. Multidimensional scaling [100] was performed with the *cmdscale* function. To test significance of groups in ordination revealed from PCA and MDS, PERMANOVA [101] was made with the *adonis2* function from the *vegan* package v. 1.6-10 [102]. Euclidean distance and 10^5^ permutations were used. Orthogonal partial least squares (OPLS-DA) was used for classification with *ropls* v. 1.32.0. Factor loadings of predictive component and variable importance in projection (VIP) were used to assess statistical relationships between features and factors of interest [103]. For metabolite set enrichment analysis, the *fgsea* algorithm v. 1.26.0 was used [104].

Metabolite sets for metabolic pathways were downloaded from the KEGG database [105] through the *KEGGREST* package v. 1.40.1 [106], with *M. truncatula* as the reference organism. Lists of metabolites for pathways were manually corrected; poorly represented or extra-large sets were excluded; for some metabolites, obligatory needful pathways were added; compounds identified up to class (hexose, disaccharide, among others) were joined to relevant pathways. Correlation graphs were built from the *Cytoscape* software v. 3.10.3 [107].

## 5. Conclusions

The effect of *R. irregularis* inoculation and phosphorus treatment on the content of 327 metabolites (amino acids, carboxylic and fatty acids, sterols, phenolic compounds and sugars) in the roots of *M. lupulina* during key stages of the host plant development, from the first leaf stage to the mature fruit, was evaluated. The results of this study showed that the highest AM efficiency in the highly efficient PMS “*M. lupulina* + *R. irregularis*” manifested itself under Pi deficiency conditions at the BI stage, and under conditions of optimal phosphorus nutrition. On the contrary, low AM efficiency (in comparison with the values for plants in the AM+P- variant) and lower mycorrhization parameters were observed for plants in the AM+P+ variant. Only the BI stage was characterized by the most significant AM-induced metabolic rearrangements in the roots of the host plant. Probably, the BI stage is key for the development of an efficient AM symbiosis. The efficient AM is accompanied by a strong increase in the metabolism of proteins, carbohydrates and lipids, as well as a significant increase in the content of phosphates (methyl phosphate, phosphoric acid). Mycorrhization generally downregulated the acid content of the TCA cycle, both under conditions of deficiency and at the optimal Pi level for nutrition. As a result of the study, among the metabolites in the roots of *M. lupulina*, the following 14 markers of the efficient AM symbiosis can be distinguished, characterized by upregulation with a deficiency and a sufficient Pi level: hexose_RI = 1881, compsug_RI = 3273, trehalose (carbohydrate metabolism), glycerol, glycerol-3P, ethanolamine phosphate, campesterol, sterol_RI = 3260, sterol_RI = 3362, myo-inositol-2P (lipid metabolism), methyl phosphate and phosphoric acid. An intensive upregulation of the phosphate content indicates that inoculation with AMF significantly leveled the effect of phosphorus deficiency on AM+P- plants. A negative marker of the efficient AM symbiosis was succinic acid (and to a lesser extent citric acid). Its content was, mainly, lower during mycorrhization, both with deficiency and with an optimal Pi level.

Based on the analysis of metabolic alterations, it can be concluded that mycorrhization led to the adaptation of plants to low Pi level conditions. AM determined the major proportion of metabolic rearrangements with different Pi availability; therefore, AM should be considered as the priority factor on the metabolome of the host plant over the phosphorus supply.

## Figures and Tables

**Table 1:** Statistics of metabolite networks (see Figure 5) from strong (*r* > 0.9) Pearsons’s correlations of their content during development under different phosphorus and mycorrhization status.

Statistics Parameters	AM+P+	AM+P-	AM-P+	AM-P-
Number of nodes	319	309	320	318
Number of edges	2778	3758	3745	1973
+	1490	2150	2195	1142
−	1288	1608	1550	831
+/−	1.2	1.3	1.4	1.4
Avg. number of neighbors	17.4	24.5	23.7	12.8
Network diameter	15	10	12	11
Network radius	8	6	6	7
Characteristic path length	4.1	3.4	3.5	4.2
Clustering coefficient	491	525	540	489
Network density	55	80	75	42
Network heterogeneity	810	765	666	735
Network centralization	113	143	110	93

## Data Availability

The original contributions presented in this study are included in the article. Further inquiries can be directed to the corresponding author.

## Supplementary Materials

The following supporting information can be downloaded at: https://www.mdpi.com/article/10.3390/plants14172685/s1, Figure S1. Differences in the profiles of plant metabolites with different mycorrhization and phosphorus status: graphs of PCA scores. The points correspond to the profiles of metabolites, % is the proportion of variance associated with the principal component (PC), ellipses are 90% intervals. Figure S2. Metabolite set enrichment analysis based on loadings from OPLS-DA classification at different vegetative stages of *M. lupulina* development. The metabolites were selected according to VIP > 1 predictive components of the OPLS_DA models. The red color corresponds to a higher level relative to AM-P+. Figure S3. Loadings of predictive components from the corresponding OPLS-DA models at the 1L stage. Figure S4. Loadings of predictive components from the corresponding OPLS-DA models at the 2L stage. Figure S5. Loadings of predictive components from the corresponding OPLS-DA models at the SI/3L stage. Figure S6. Loadings of predictive components from the corresponding OPLS-DA models at the BI stage. Figure S7. Metabolite set enrichment analysis based on loadings from OPLS-DA classification at different generative stages of *M. lupulina* development. The metabolites were selected according to VIP > 1 predictive components of the OPLS_DA models. The red color corresponds to a higher level relative to AM-P+. Figure S8. Loadings of predictive components from the corresponding OPLS-DA models at the FL stage. Figure S9. Loadings of predictive components from the corresponding OPLS-DA models at the MF stage. Figure S10. Key metabolic rearrangements in AM+P+, AM+P-, AM-P- vs. AM-P+ plants: stages of *M. lupulina* development (A), key metabolic processes and marker metabolites involved in the development of effective AM symbiosis (B). “+”—upregulation (vs. AM-P+); “-“—downregulation vs. AM-P+; empty cells—absence of significant (*p* > 0.05) differences in the compared variants; TCA—tricarboxylic acid. Table S1. PERMANOVA for the data in the Figure S1.
