# Supplementary material for: The Effect of Arbuscular Mycorrhizal Fungus and Phosphorus Treatment on Root Metabolome of Medicago lupulina During Key Stages of Development
Source: Plants (Basel). 2025 Aug 28;14(17):2685. doi: 10.3390/plants14172685 (PMC12430555; doi:10.3390/plants14172685)
Supplement: Supplementary file 1 [file plants-14-02685-s001.zip › Supplementary Material Captions.pdf]

**Supplementary Materials:**

Yurkov, A.P.; Puzanskiy, R.K.; Kryukov, A.A.; Kudriashova, T.R.; Kovalchuk, A.I.; Gorenkova, A.I.; Bogdanova, E.M.; Laktionov, Y.V.; Romanyuk, D.A.; Yemelyanov, V.V.; Shavarda A.L.; Shishova M.F. The Effect of Arbuscular Mycorrhizal Fungus and Phosphorus Treatment on Root Metabolome of *Medicago lupulina* During Key Stages of Development. *Plants* **2025**, *14*, 2685.

**Figure S1.** Differences in the profiles of plant metabolites with different mycorrhization and phosphorus status: graphs of PCA scores. The points correspond to the profiles of metabolites, % is the proportion of variance associated with the principal component (PC), ellipses are 90% intervals.

**Figure S2.** Metabolite set enrichment analysis based on loadings from OPLS-DA classification at different vegetative stages of *M. lupulina* development. The metabolites were selected according to VIP>1 predictive components of the OPLS_DA models. The red color corresponds to a higher level relative to AM-P+.

**Figure S3.** Loadings of predictive components from the corresponding OPLS-DA models at the 1L stage.

**Figure S4.** Loadings of predictive components from the corresponding OPLS-DA models at the 2L stage.

**Figure S5.** Loadings of predictive components from the corresponding OPLS-DA models at the SI/3L stage.

**Figure S6.** Loadings of predictive components from the corresponding OPLS-DA models at the BI stage.

**Figure S7.** Metabolite set enrichment analysis based on loadings from OPLS-DA classification at different generative stages of *M. lupulina* development. The metabolites were selected according to VIP>1 predictive components of the OPLS_DA models. The red color corresponds to a higher level relative to AM-P+.

**Figure S8.** Loadings of predictive components from the corresponding OPLS-DA models at the FL stage.

**Figure S9.** Loadings of predictive components from the corresponding OPLS-DA models at the MF stage.

**Figure S10.** Key metabolic rearrangements in AM+P+, AM+P-, AM-P- vs AM-P+ plants: stages of *M. lupulina* development (A), key metabolic processes and marker metabolites involved in the development of effective AM symbiosis (B). "+" – upregulation (vs AM-P+); "-" – downregulation vs AM-P+; empty cells – absence of significant (P>0.05) differences in the compared variants; TCA – tricarboxylic acids.

**Table S1.** PERMANOVA for the data in the Figure S1.
