# Supplementary material for: The Effect of Arbuscular Mycorrhizal Fungus and Phosphorus Treatment on Root Metabolome of Medicago lupulina During Key Stages of Development
Source: Plants (Basel). 2025 Aug 28;14(17):2685. doi: 10.3390/plants14172685 (PMC12430555; doi:10.3390/plants14172685)
Supplement: Supplementary file 1 [file plants-14-02685-s001.zip › Supplementary Materials.pdf]

Supplementary Materials:

Yurkov, A.P.; Puzanskiy, R.K.; Kryukov, A.A.; Kudriashova, T.R.; Kovalchuk, A.I.; Gorenkova, A.I.; Bogdanova, E.M.; Laktionov, Y.V.; Romanyuk, D.A.; Yemelyanov, V.V.; Shavarda A.L.; Shishova M.F. The Effect of Arbuscular Mycorrhizal Fungus and Phosphorus Treatment on Root Metabolome of *Medicago lupulina* During Key Stages of Development. *Plants* **2025**, *14*, 2685.

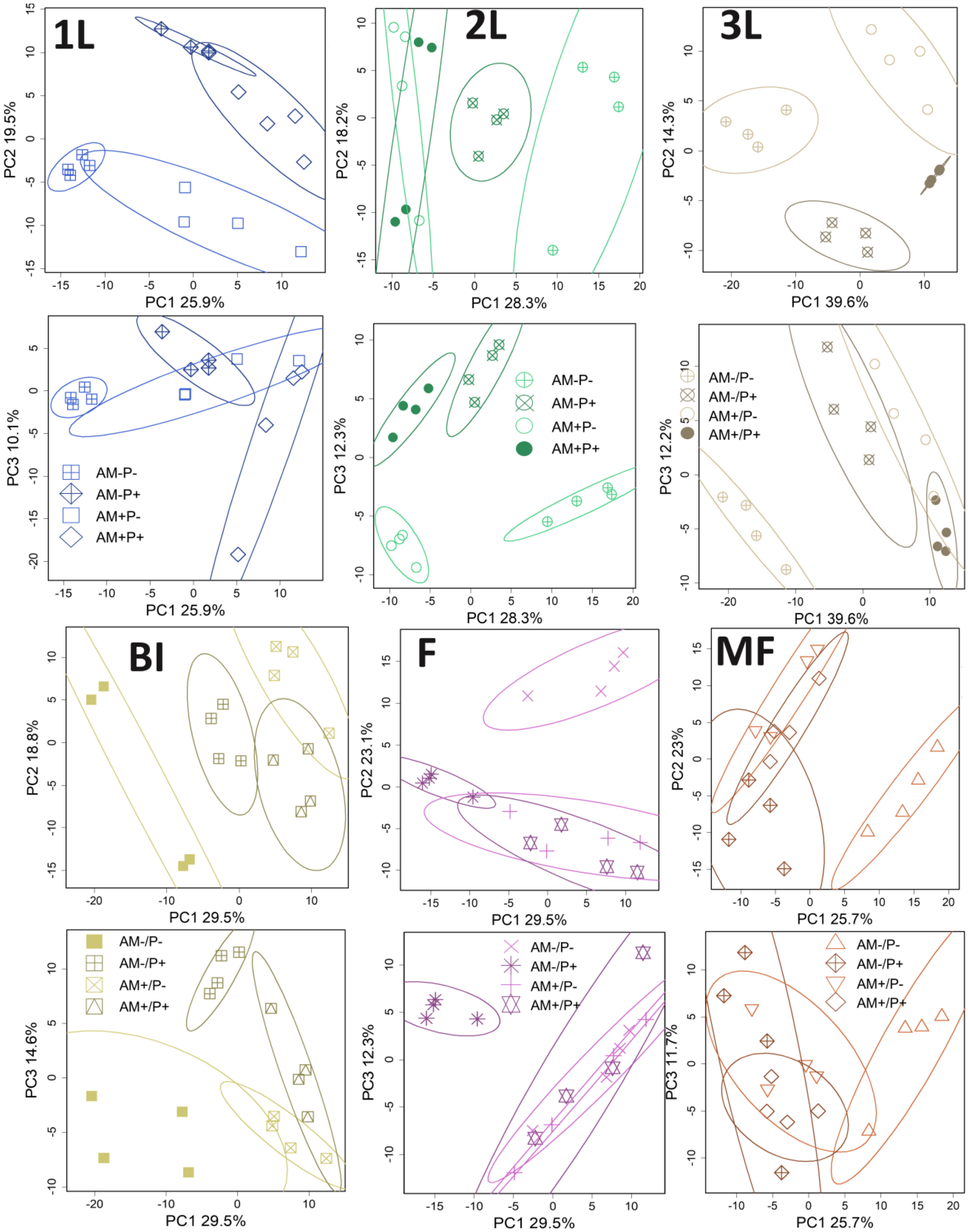

Figure S1. Differences in the profiles of plant metabolites with different mycorrhization and phosphorus status: graphs of PCA scores. The points correspond to the profiles of metabolites, % is the proportion of variance associated with the principal component (PC), ellipses are 90% intervals.

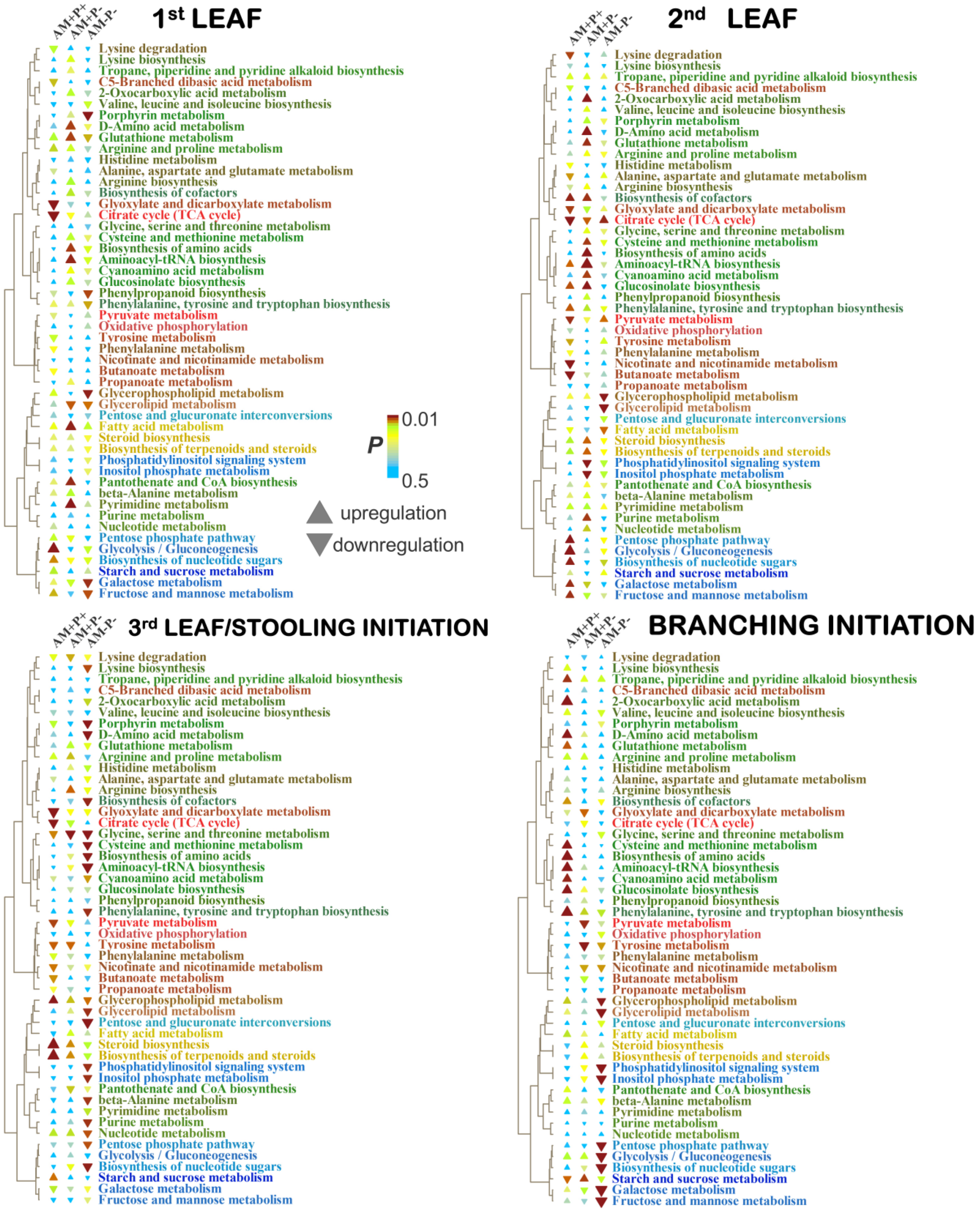

Figure S2. Metabolite set enrichment analysis based on loadings from OPLS-DA classification at different vegetative stages of *M. lupulina* development. The metabolites were selected according to VIP>1 predictive components of the OPLS_DA models. The red color corresponds to a higher level relative to AM-P+.

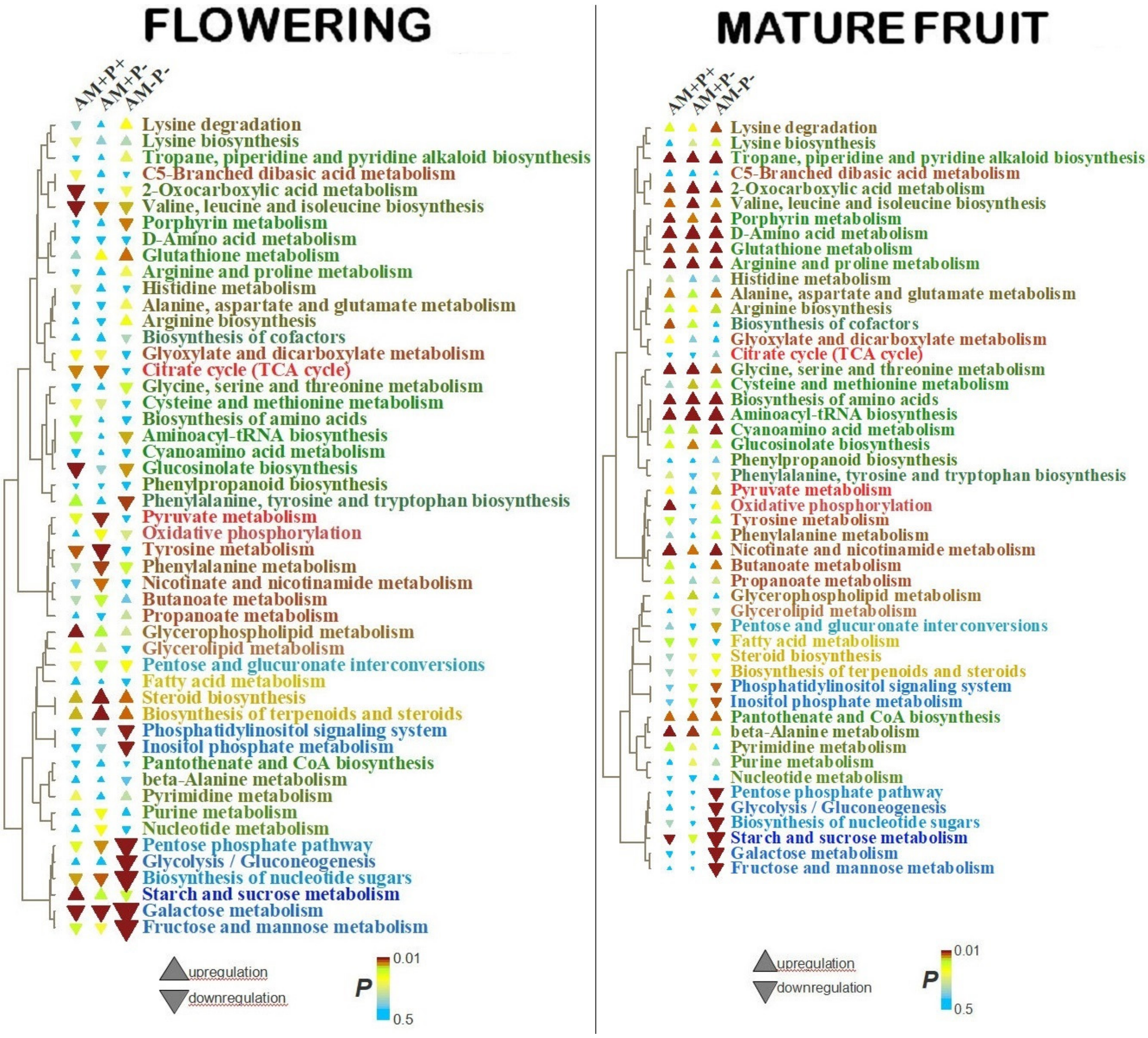

Figure S3. Loadings of predictive components from the corresponding OPLS-DA models at the 1L stage.

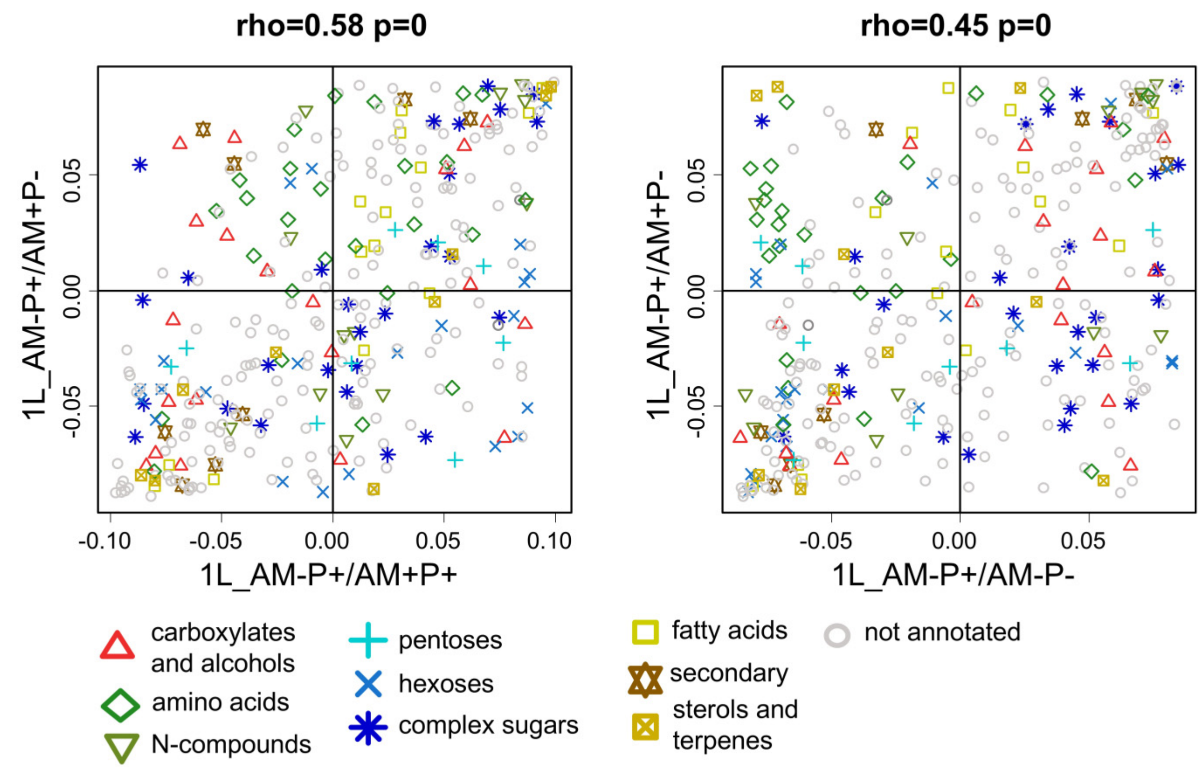

Figure S4. Loadings of predictive components from the corresponding OPLS-DA models at the 2L stage.

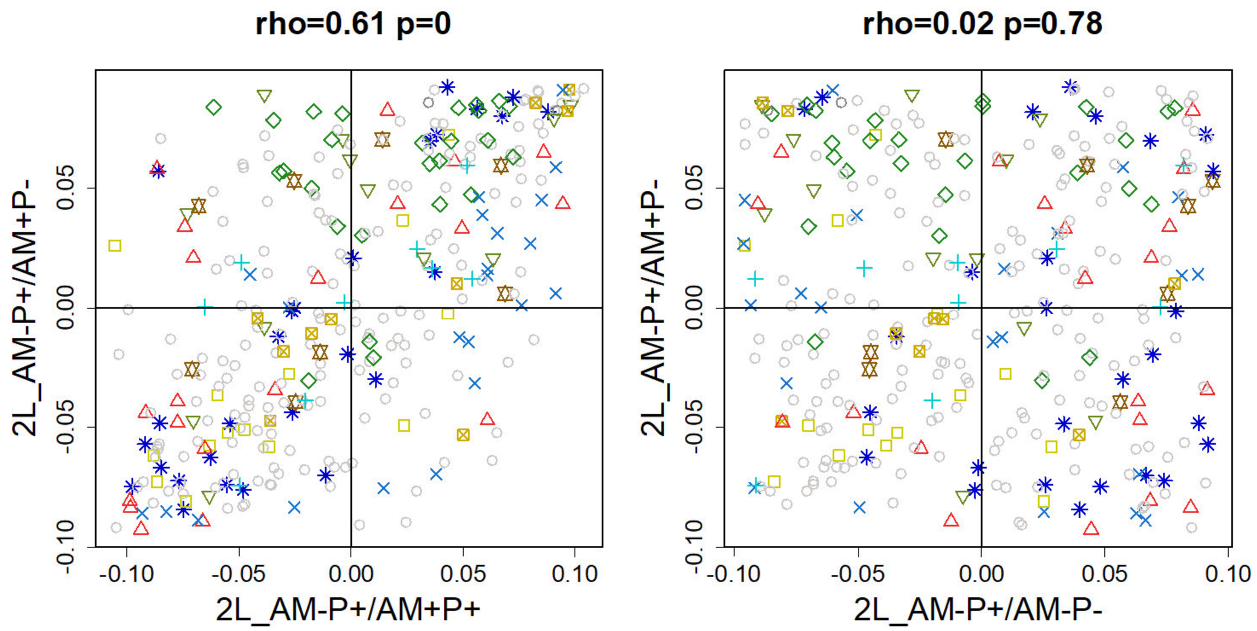

Figure S5. Loadings of predictive components from the corresponding OPLS-DA models at the SI/3L stage.

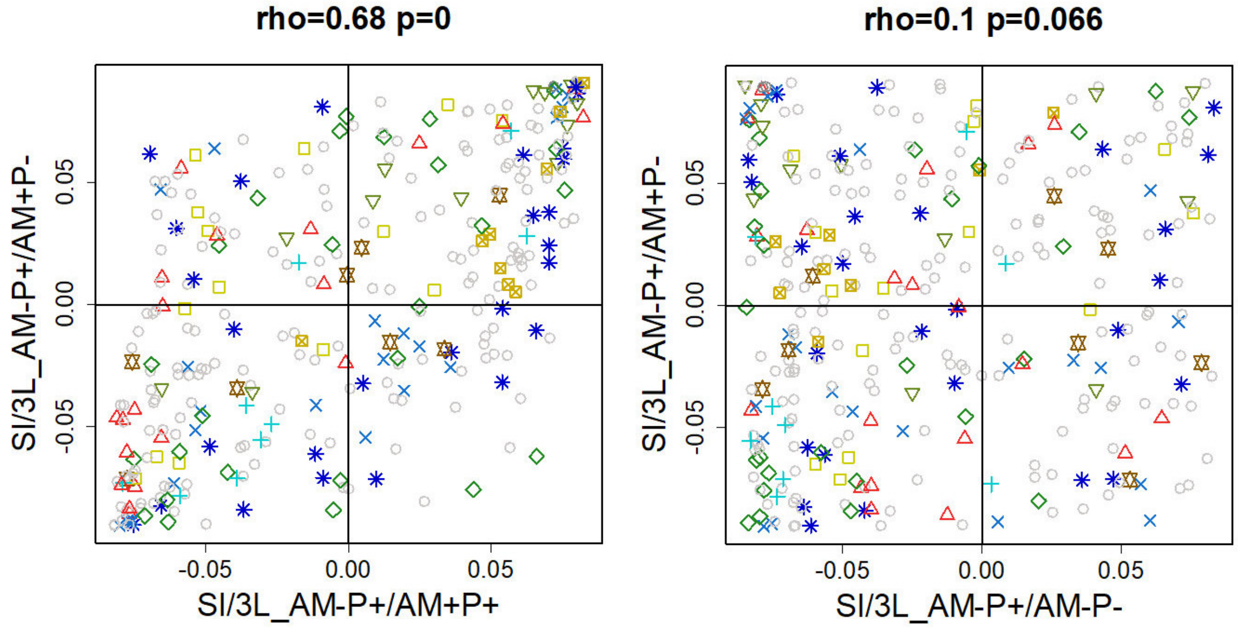

Figure S6. Loadings of predictive components from the corresponding OPLS-DA models at the BI stage.

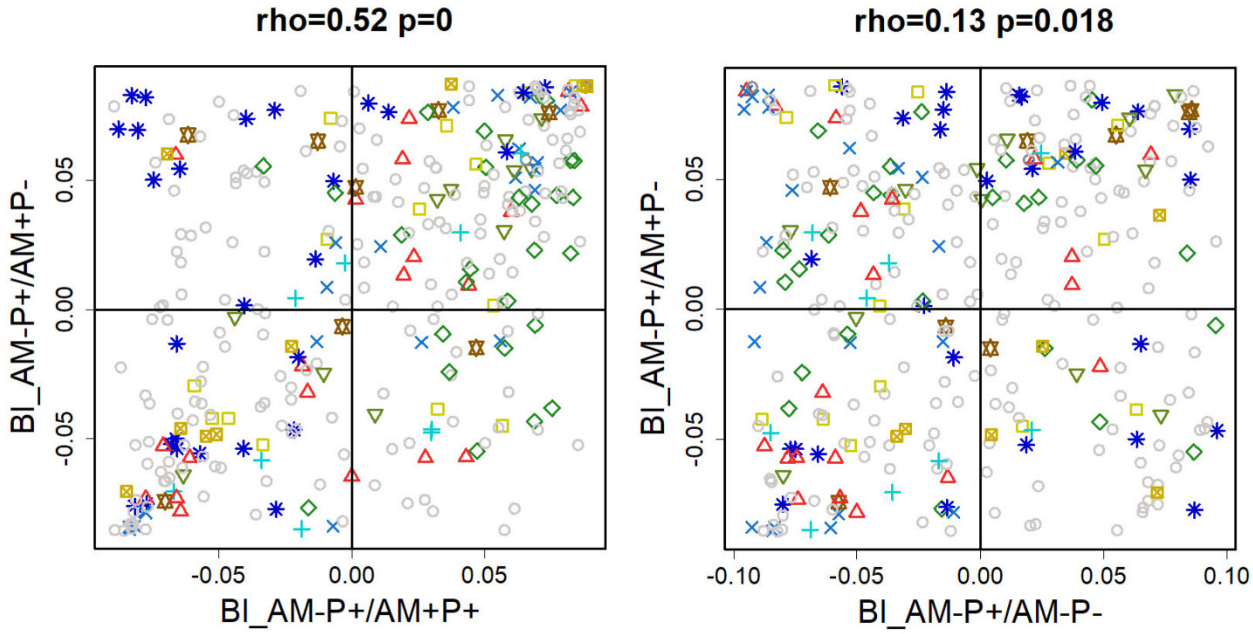

Figure S7. Metabolite set enrichment analysis based on loadings from OPLS-DA classification at different generative stages of *M. lupulina* development. The metabolites were selected according to VIP>1 predictive components of the OPLS_DA models. The red color corresponds to a higher level relative to AM-P+.

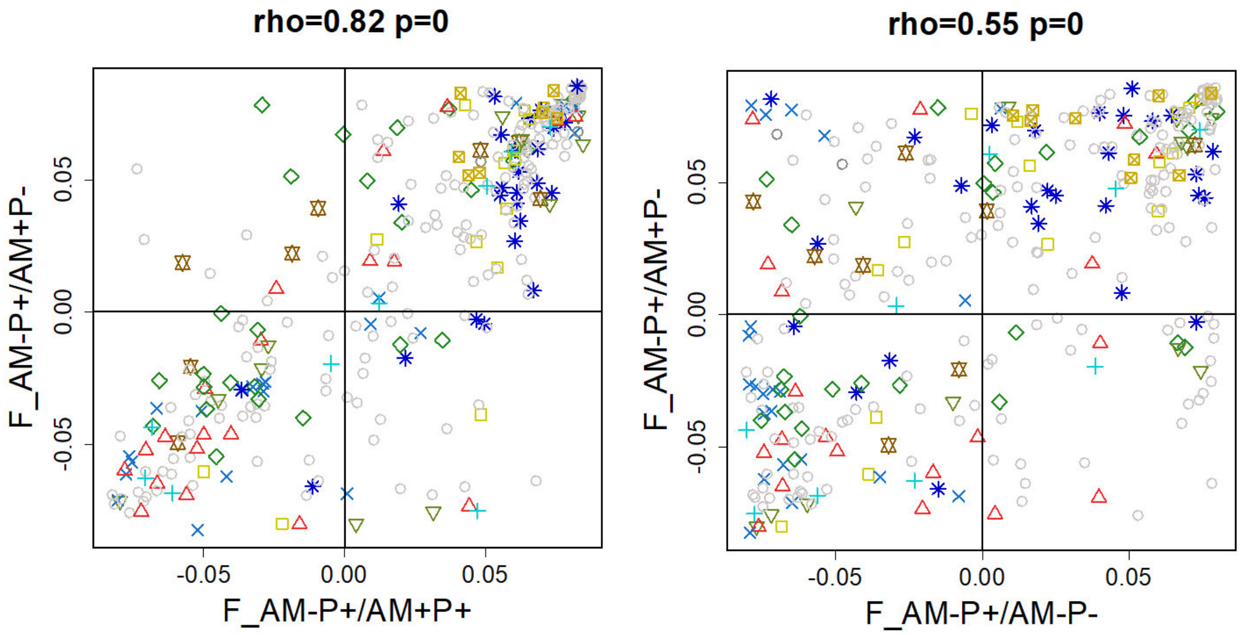

Figure S8. Loadings of predictive components from the corresponding OPLS-DA models at the FL stage.

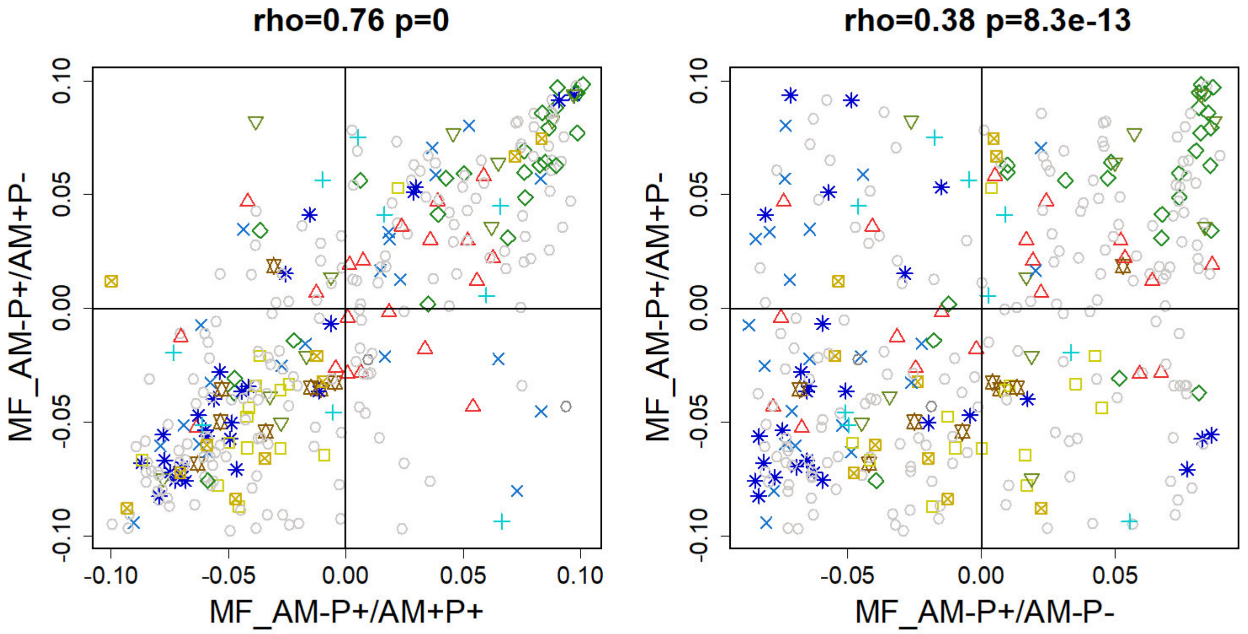

Figure S9. Loadings of predictive components from the corresponding OPLS-DA models at the MF stage.

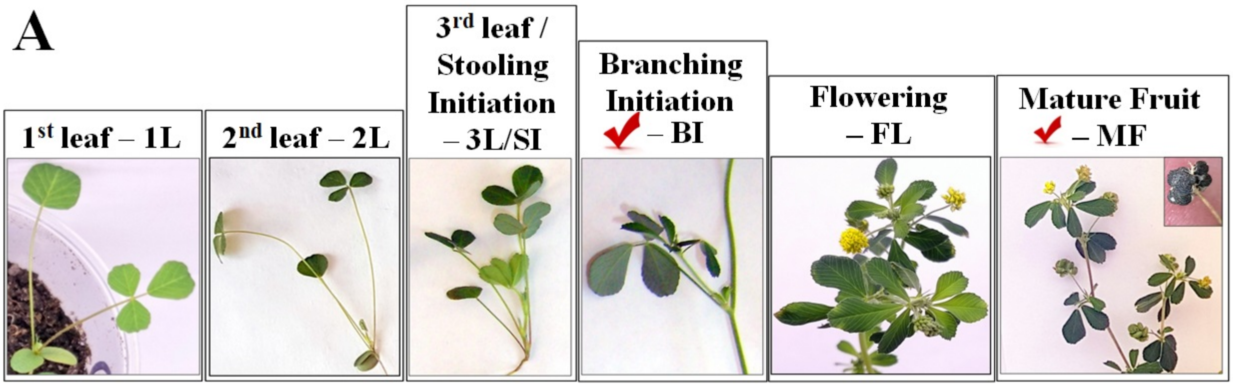

B

Metabolic processes	Main process	AM+P+ vs AM-P+						AM+P- vs AM-P+						AM-P- vs AM-P+					
		1L	2L	SI	BI	FL	MF	1L	2L	SI	BI	FL	MF	1L	2L	SI	BI	FL	MF
Aminoacyl-tRNA biosynthesis	Protein biosynthesis		+		+	-	+	+	+	-	+		+	-	-	-		-	+
Biosynthesis of cofactors	Protein biosynthesis		+		+		+	+	+	-			+	-	-	-	-	-	
Valine, leucine and isoleucine biosynthesis	Protein biosynthesis		-	-	+	-	+		+			-	+	-	+		-	-	+
Arginine biosynthesis	Protein biosynthesis		-		+		+	+	+	+			+			-	+	+	+
Biosynthesis of amino acids	Protein biosynthesis				+	-	+	+	+	-			+	-		-			+
Phenylalanine, tyrosine and tryptophan biosynthesis	Protein biosynthesis	+	+		+	+	+	+	+		+			-	-	-	-	-	-
beta-Alanine metabolism	Protein biosynthesis	+	+		+		+	+	+		+		+			-	-		+
Alanine, aspartate and glutamate metabolism	Protein biosynthesis	-	-	-	+		+						+		+	-		+	+
Cyanoamino acid metabolism	Protein biosynthesis			-	+		+	+	+	-			+	-		-			+
Starch and sucrose metabolism	Carbohydrate metabolism	+	+	+	-	+	-				+	+	-	+	+		-	-	-
Galactose metabolism	Carbohydrate metabolism	+	+	-	+	-		-	-	-	-	-		-		-	-	-	-
Fructose and mannose metabolism	Carbohydrate metabolism	+	+		+	-			-		-	-		-	-	-	-	-	-
Glycerolipid metabolism	Lipid metabolism				+	+		-		+	+	+	-	-	-	-	-		-
Glycerophospholipid metabolism	Lipid metabolism	+	+	+	+	+	+		+	+	+	+	+	-	-	-	-	+	
Fatty acid metabolism	Lipid metabolism	+	-		+		-	+		+	+		-	+	-	+			
Biosynthesis of terpenoids and steroids	Lipid metabolism.	+	+	+		+	-	+	+	+	+	+	-	-	-	-	+	+	-
Glycolysis / Gluconeogenesis	Energy exchange	+	+		+					+	+			-	-		-	-	-
Pyruvate metabolism	Energy exchange	-	-	-		-	+		-	-	-	-		+	+		-		+
Pantothenate and CoA biosynthesis	Energy exchange	+	+	-	+		+	+	+	-			+		-	-			+
Citrate cycle (TCA cycle)	Energy exchange	-	-	-		-		-	-	-	-	-	-		+			-	
Glucosinolate biosynthesis	Protective reactions		+		+	-	+	+	+	-	+	-	+	-		-	-	-	+
Glutathione metabolism	Protective reactions	+			+	+	+	+	+	+		+	+	-	-	-		+	+
Glyoxylate and dicarboxylate metabolism	Protective reactions	-	-	-	-	-	+		-	-	-	-			+	-			

Metabolites	Main process	AM+P+ vs AM-P+						AM+P- vs AM-P+						AM-P- vs AM-P+					
		1L	2L	SI	BI	FL	MF	1L	2L	SI	BI	FL	MF	1L	2L	SI	BI	FL	MF
hexose_RI=1881	Carbohydrate metabolism	+	+	+		+		+	+	+	+	+	+	+	-	-	-	-	-
compsug_RI=3273	Carbohydrate metabolism	+	+	+		+		+	+	+	+	+					+		-
Trehalose	Carbohydrate metabolism		+	+	+	+	+	+	+	+	+	+	+	-	-		-	-	-
Glycerol	Lipid metabolism	+	-			-		+	-		+					-	-	-	
Glycerol-3P	Lipid metabolism	+	-	+	+	+			-	+	+	+		-	-	-	-		
Ethanolaminephosphate	Lipid metabolism	+	+	+	+	+			+	+	+			-	-	-		+	
Campesterol	Lipid metabolism	+	+	+	+	+		+	+	+	+	+							
sterol_RI=3260	Lipid metabolism	+	+	+	+	+		+	+	+	+	+		-	-				
sterol_RI=3362	Lipid metabolism	+	+			+	+	+	+		+	+	+	-	-	-			
myo-Inositol-2P	Lipid metabolism	+	+	+	+	+	+			+	+	+		-	-	-	-	-	-
Methyl phosphate	Energy exchange	+		+	+				+	+	+	+		-	-	-	-		
Phosphoric acid	Energy exchange	+	+	+	+	+	+		+	+	+	+			-	-	-	-	
Citric acid (citrate) content in TCA cycle	Energy exchange	-		-				-						+	+	+			
Succinic acid (succinate) content in TCA cycle	Energy exchange	-	-	-	-	-			-	-	-	-		+	+				+

Figure S10. Key metabolic rearrangements in AM+P+, AM+P-, AM-P- vs AM-P+ plants: stages of *M. lupulina* development (A), key metabolic processes and marker metabolites involved in the development of effective AM symbiosis (B). "+" – upregulation (vs AM-P+); "-" – downregulation vs AM-P+; empty cells – absence of significant (P>0.05) differences in the compared variants; TCA – tricarboxylic acids.

Table S1. PERMANOVA for the data in the Figure S1.

Figure S1 (PC1-3): plant stage	*p*
1L	$p \leq 0.00001$
2L	$p \leq 0.00001$
SI/3L	$p \leq 0.00001$
BI	$p \leq 0.00001$
F	$p = 0.00002$
MF	$p = 0.00002$

Note: "1L" – 1st Leaf, "2L" – 2nd Leaf, "SI/3L" – 3rd Leaf / Stooling Initiation; "BI" – Branching Initiation; "F" – Flowering; "MF" – Mature Fruit.
