# Supplementary figures and images for: The Effect of Arbuscular Mycorrhizal Fungus and Phosphorus Treatment on Root Metabolome of Medicago lupulina During Key Stages of Development
Source: Plants (Basel). 2025 Aug 28;14(17):2685. doi: 10.3390/plants14172685 (PMC12430555; doi:10.3390/plants14172685)

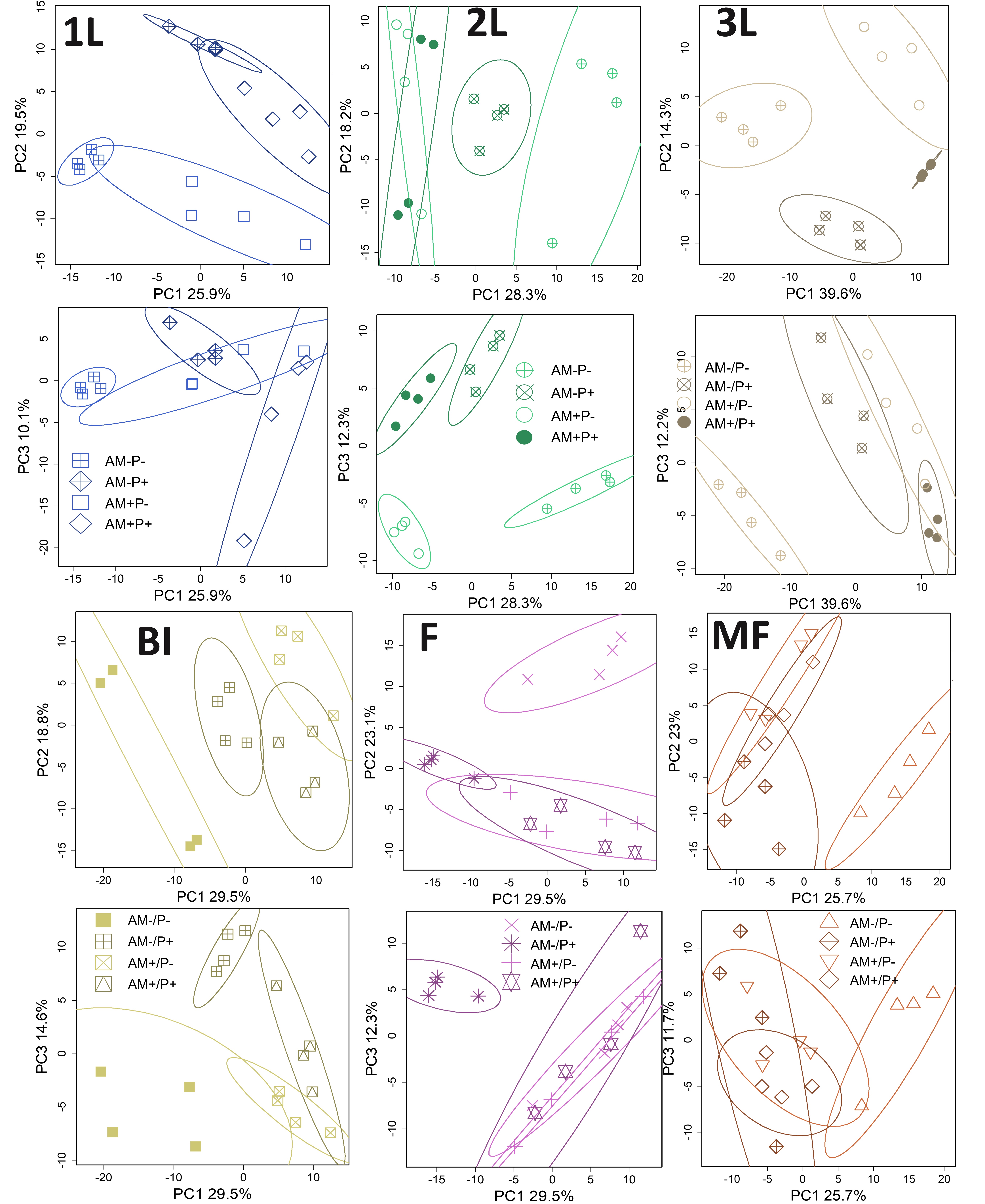

Supplement: Supplementary file 1 [file plants-14-02685-s001.zip › Figure S1.jpg]

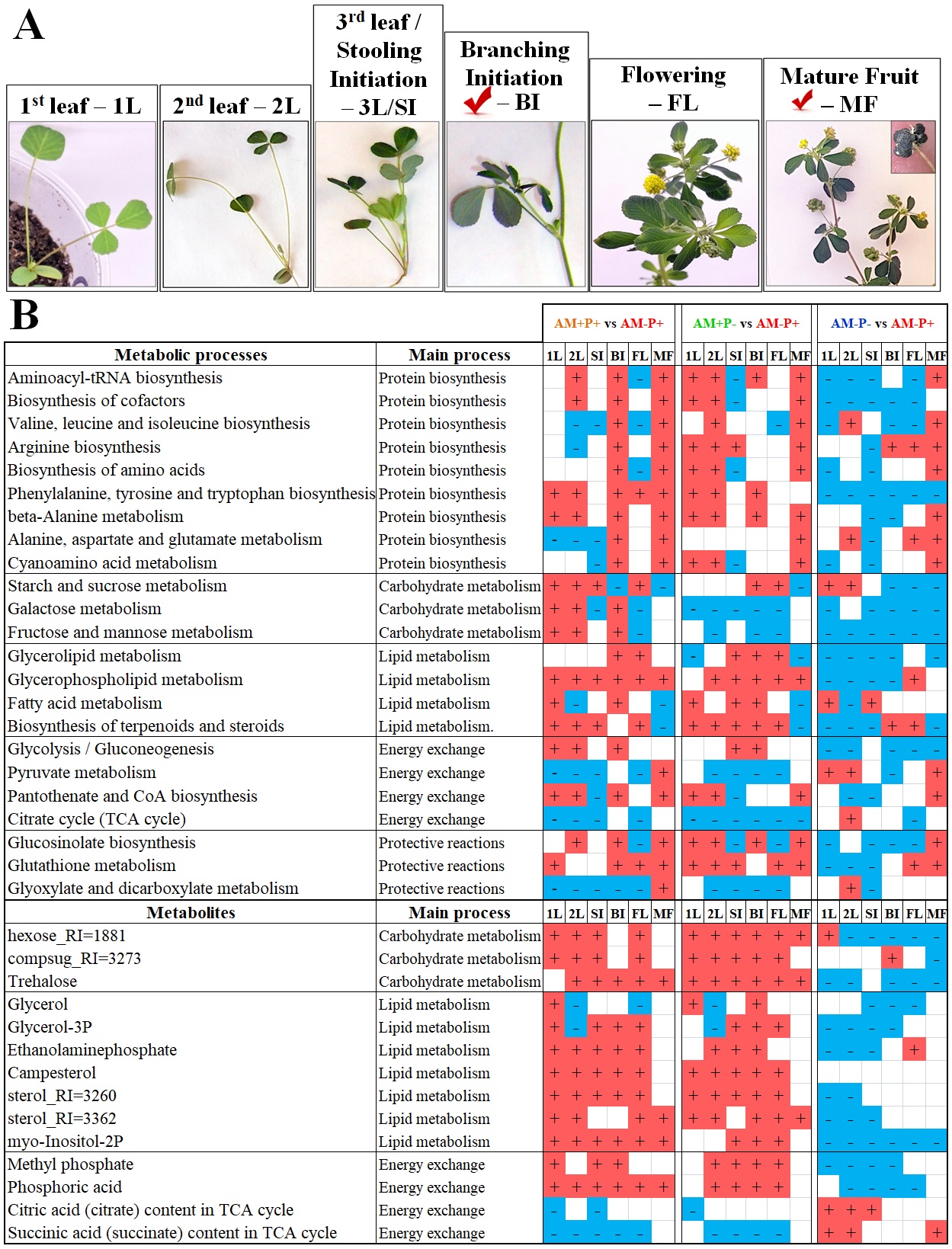

Supplement: Supplementary file 1 [file plants-14-02685-s001.zip › Figure S10.jpg]

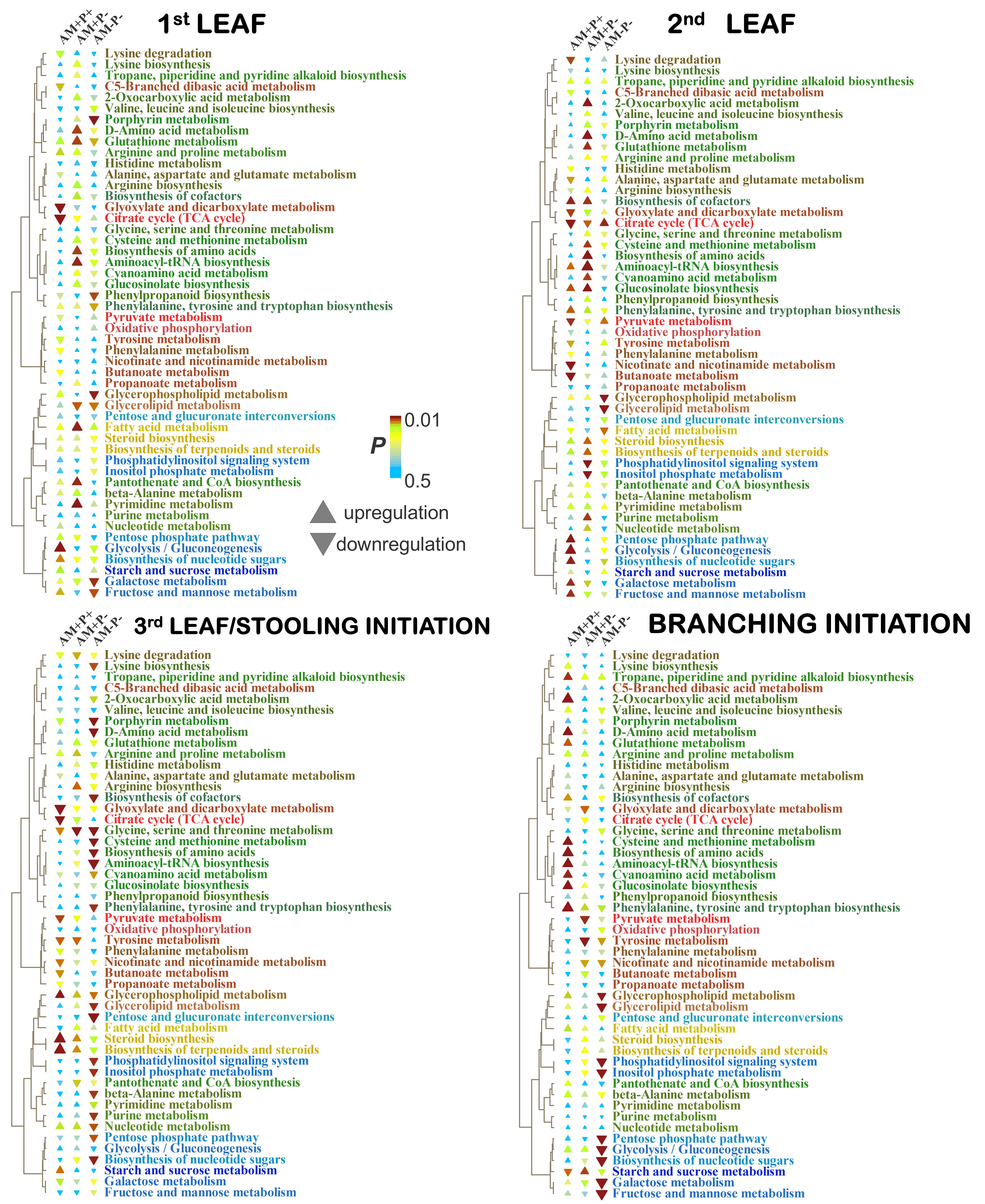

Supplement: Supplementary file 1 [file plants-14-02685-s001.zip › Figure S2.jpg]

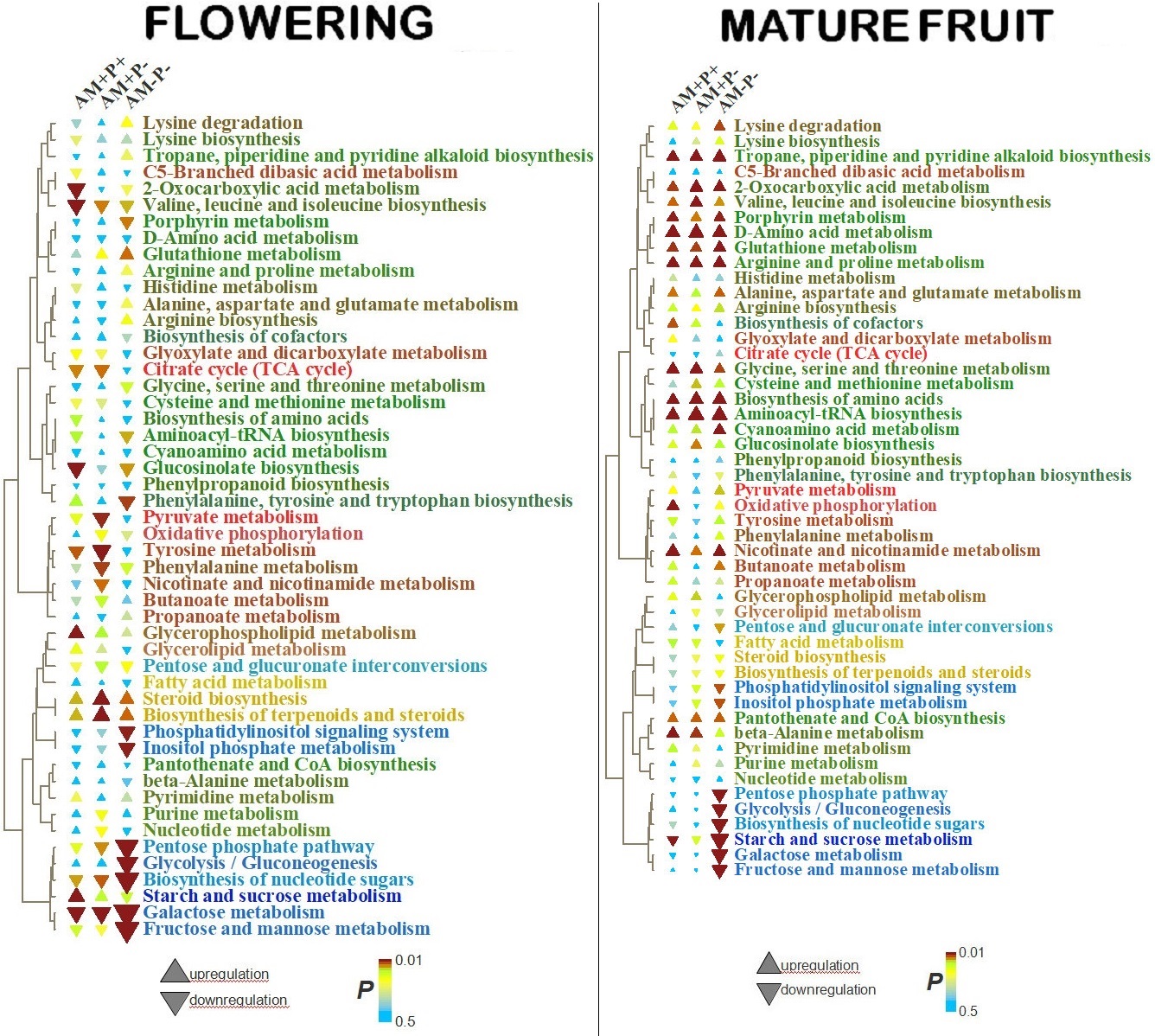

Supplement: Supplementary file 1 [file plants-14-02685-s001.zip › Figure S3.jpg]

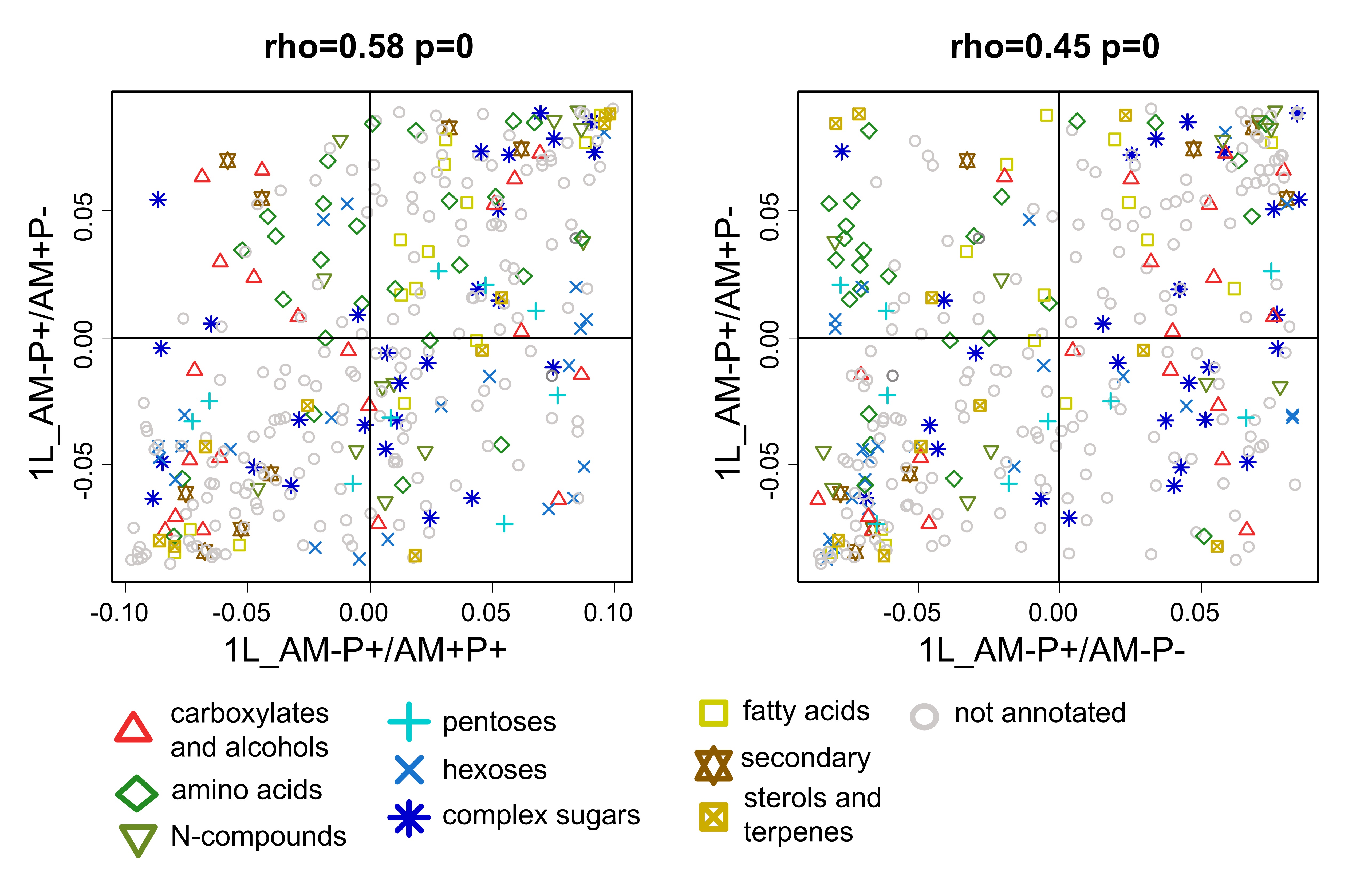

Supplement: Supplementary file 1 [file plants-14-02685-s001.zip › Figure S4.jpg]

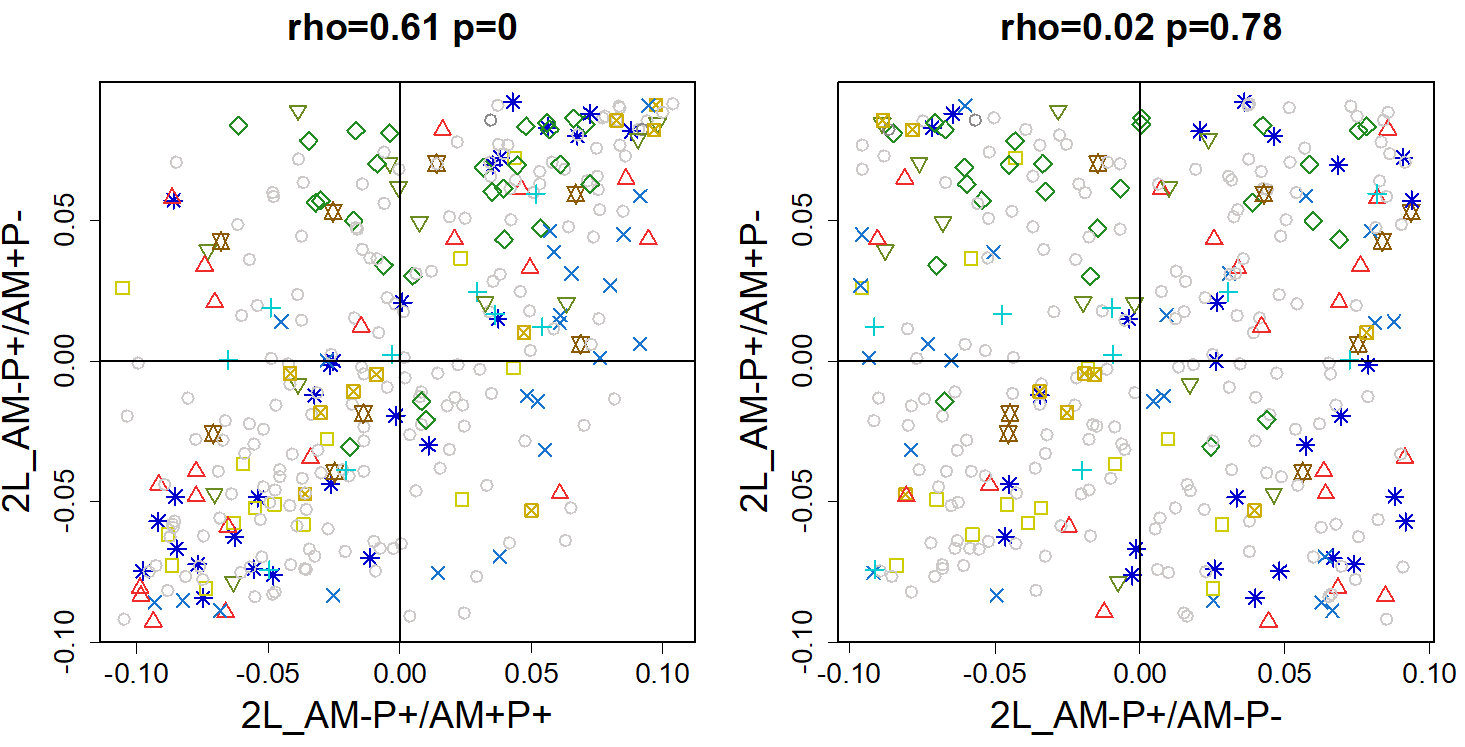

Supplement: Supplementary file 1 [file plants-14-02685-s001.zip › Figure S5.jpg]

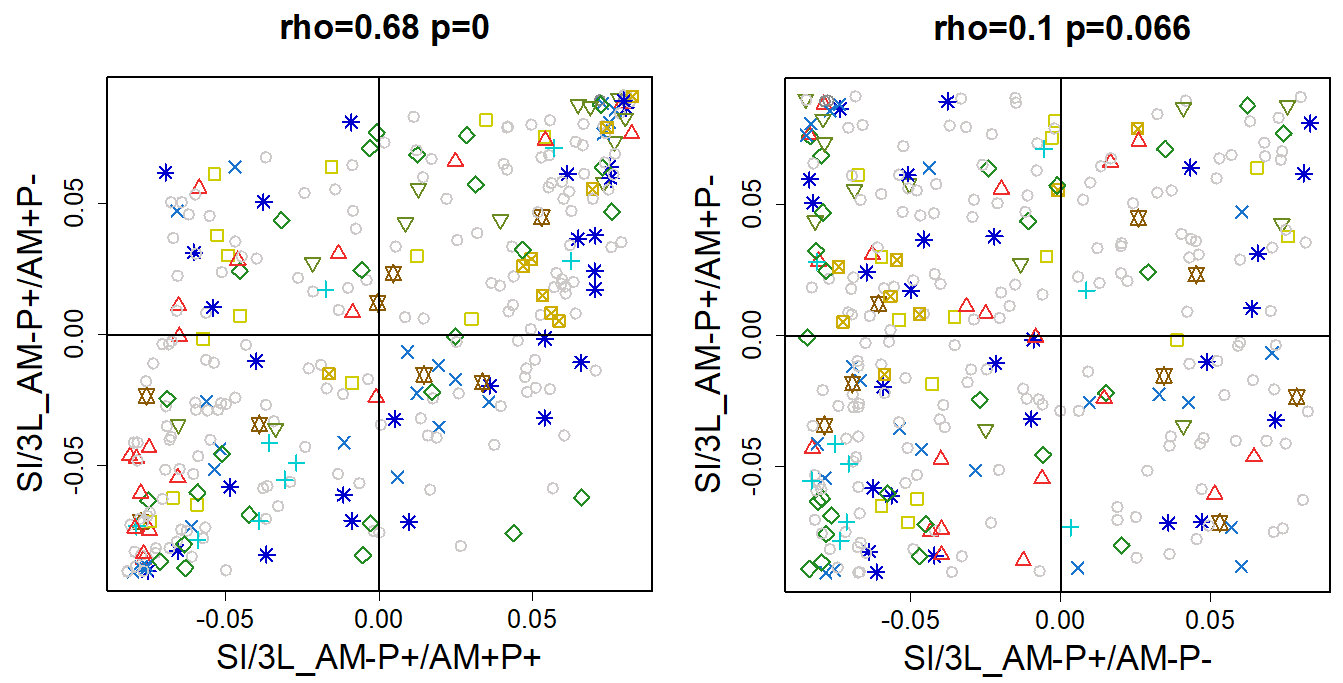

Supplement: Supplementary file 1 [file plants-14-02685-s001.zip › Figure S6.jpg]

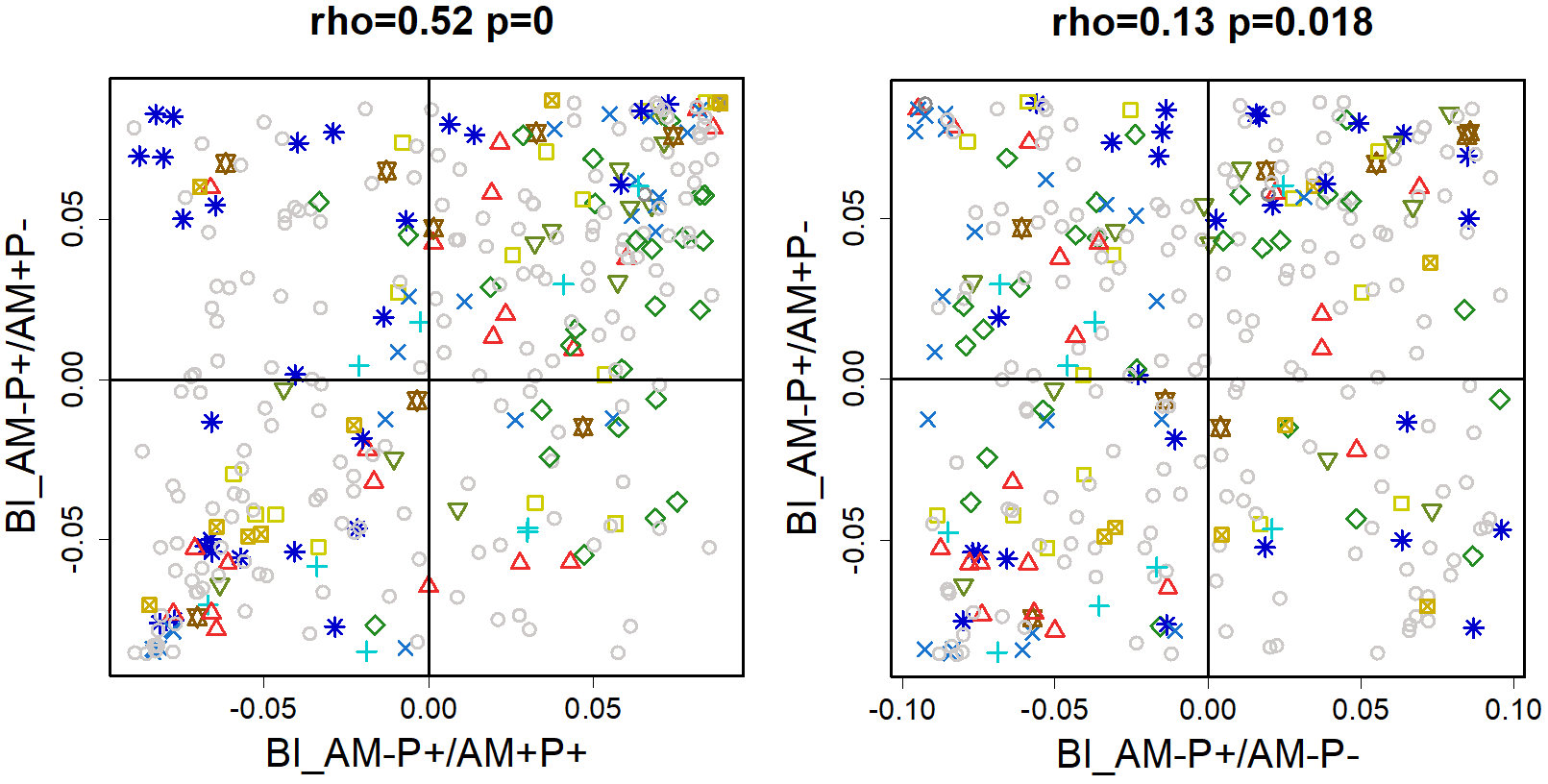

Supplement: Supplementary file 1 [file plants-14-02685-s001.zip › Figure S7.jpg]

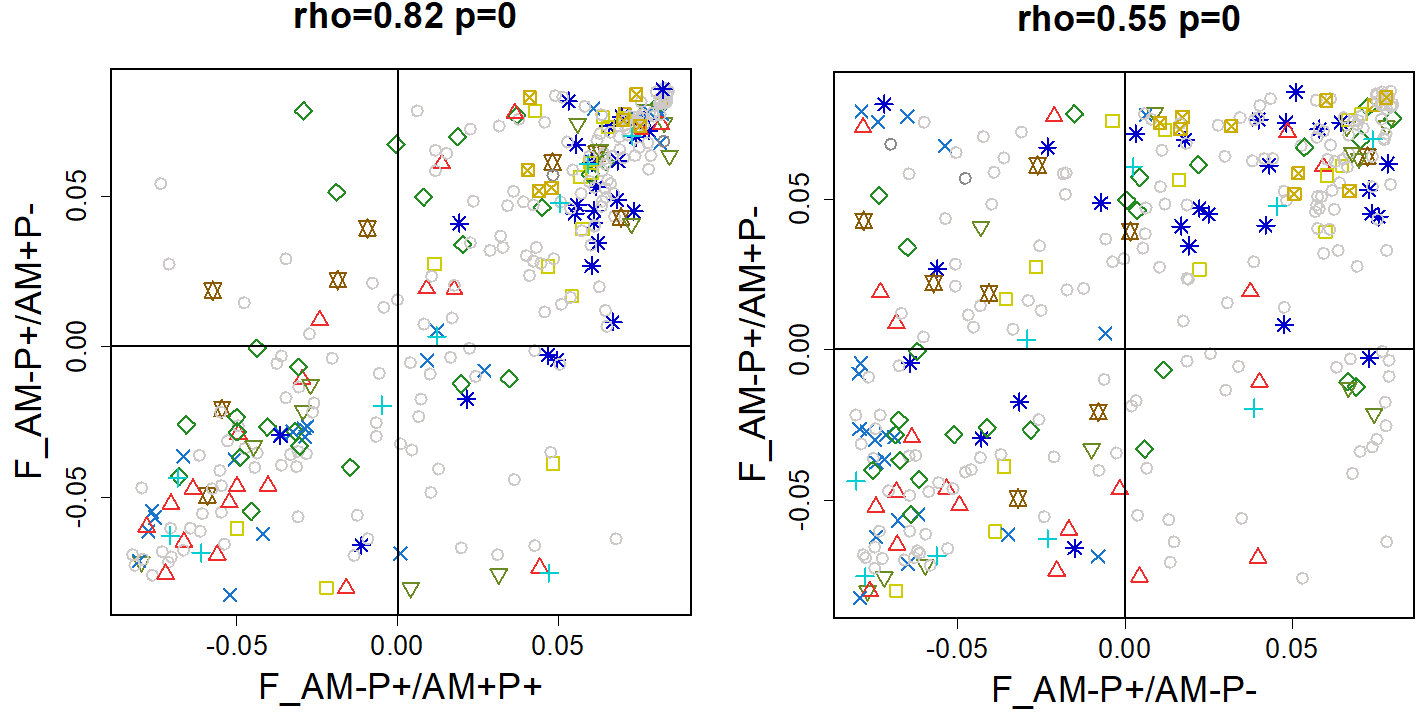

Supplement: Supplementary file 1 [file plants-14-02685-s001.zip › Figure S8.jpg]

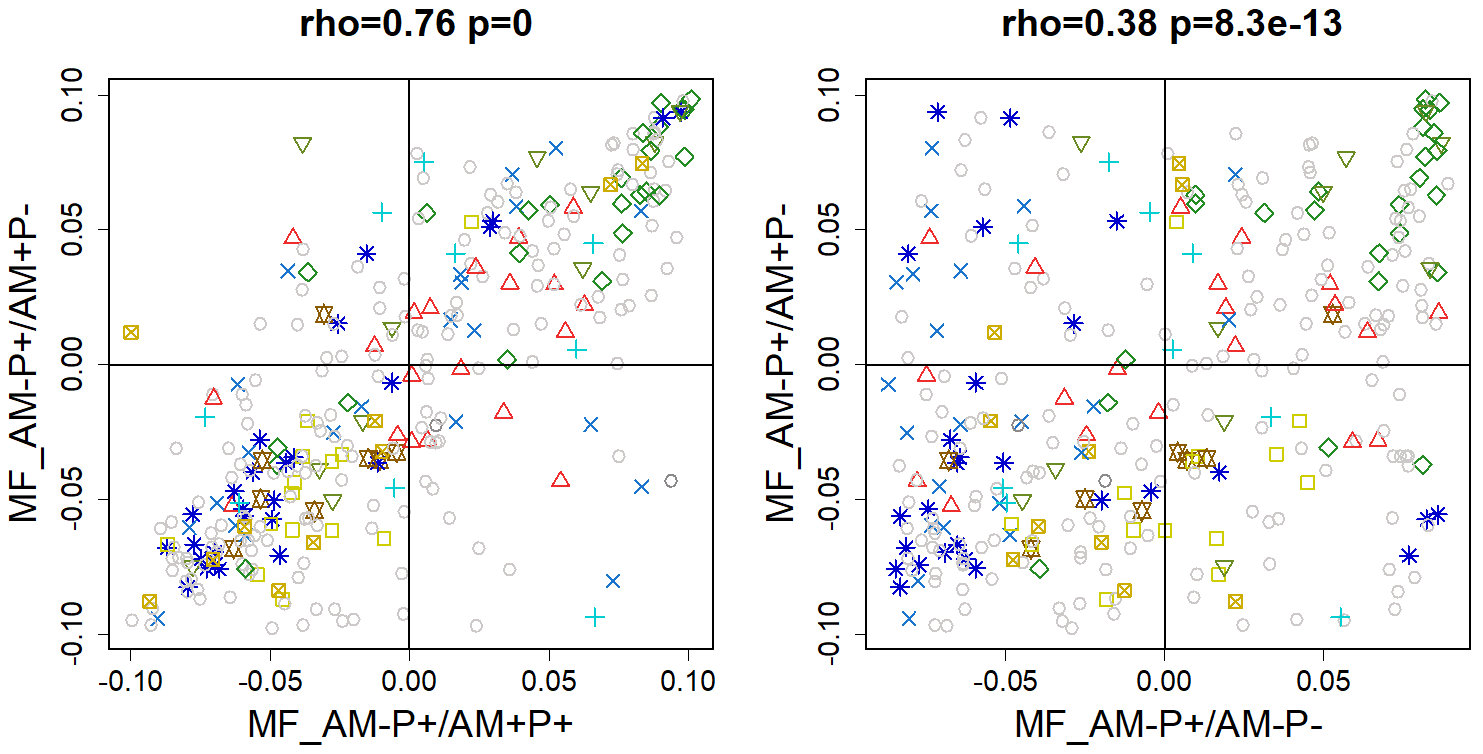

Supplement: Supplementary file 1 [file plants-14-02685-s001.zip › Figure S9.jpg]
